# Synthesis and Applications of Cinchona Squaramide‐Modified Poly(Glycidyl Methacrylate) Microspheres as Recyclable Polymer‐Grafted Enantioselective Organocatalysts

**DOI:** 10.1002/chem.202001993

**Published:** 2020-09-23

**Authors:** Sándor Nagy, Zsuzsanna Fehér, Levente Kárpáti, Péter Bagi, Péter Kisszékelyi, Béla Koczka, Péter Huszthy, Béla Pukánszky, József Kupai

**Affiliations:** ^1^ Department of Organic Chemistry & Technology Budapest University of Technology & Economics Szent Gellért tér 4 1111 Budapest Hungary; ^2^ Laboratory of Plastics & Rubber Technology Budapest University of Technology & Economics Műegyetem rkp. 3. Budapest 1111 Hungary; ^3^ Downstream Hungary Polyolefin R&D, MOL Plc. Olajmunkás utca 2 2443 Százhalombatta Hungary; ^4^ Department of Inorganic and Analytical Chemistry Budapest University of Technology & Economics Szent Gellért tér 4 1111 Budapest Hungary

**Keywords:** immobilization, Michael addition, organocatalysis, polymers, recycling

## Abstract

This work presents the immobilization of cinchona squaramide organocatalysts on poly(glycidyl methacrylate) solid supports. Preparation of the well‐defined monodisperse polymer microspheres was facilitated by comprehensive parameter optimization. By exploiting the reactive epoxy groups of the polymer support, three amino‐functionalized cinchona derivatives were immobilized on this carrier. To explore the effect of the amino linker, these structurally varied precatalysts were synthesized by modifying the cinchona skeleton at different positions. The catalytic activities of the immobilized organocatalysts were tested in the Michael addition of pentane‐2,4‐dione and *trans*‐β‐nitrostyrene with excellent yields (up to 98 %) and enantioselectivities (up to 96 % *ee*). Finally, the catalysts were easily recovered five times by centrifugation without loss of activity.

## Graphical abstract

**Squaramides on spheres**: On poly(glycidyl methacrylate) (PGMA), a well‐defined monodisperse microspherical polymer, new cinchona squaramides were immobilized by using different linkers. Their catalytic activity was tested in the Michael addition of pentane‐2,4‐dione and *trans*‐β‐nitrostyrene with excellent yields (up to 98 %) and *ee* (up to 96 % *ee*). The catalysts were recovered by centrifugation five times without loss of their activity.

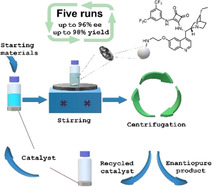

## Introduction

Nowadays, catalytic asymmetric synthesis has a growing importance, as the pharmaceutical and pesticide industries are required to produce enantiopure products.^1^ Transition metals, enzymes, and organocatalysts are the primarily used catalysts for asymmetric syntheses. Among organocatalysts, the cinchona skeleton is a widely used building block, because its numerous chiral centers and basic character have already been proven to be advantageous for asymmetric transformations.^2^ A covalently bonded dual hydrogen bond donor moiety, such as a squaramide, can further increase the catalytic efficiency of cinchonas by forming additional hydrogen bonds between the catalyst and the substrate during the catalytic process.^3^

Cinchona squaramides have found extensive application in several asymmetric catalytic reactions with high yields and selectivities, consequently their immobilization on a solid carrier is relevant. Such reactions include Michael reactions,^4^ conjugate additions,4d, 5 Mannich reactions,^6^ aza‐Henry reactions,^7^ and cycloadditions.^8^ Even though there are a few examples of homogeneous organocatalyst recycling,^9^ this topic remains challenging, and there is a considerable need for new solutions. In contrast, immobilization of the catalysts on solid supports provides simple recycling methodologies such as centrifugation,^10^ filtration,^11^ or—in case of magnetic nanoparticle support—decantation,^12^ although binding the homogeneous catalyst to a solid surface often leads to deterioration of catalytic activity.^13^

Several methods are applied for heterogenizing organocatalysts such as immobilization on non‐soluble carrier,^14^ or (co‐)polymerization.^15^ Placing an appropriate functional group at the catalyst is a simple method to form a covalent bond with the solid support through superficial functional groups. Poly(glycidyl methacrylate) (PGMA) is a polymer containing epoxy groups, therefore it is easy to functionalize with a catalyst containing primary amino group. Its preparation by dispersion radical polymerization is feasible by using glycidyl methacrylate (GMA), from which PGMA is obtained in the form of microspheres.^16^ The physicochemical properties of this polymer can be predicted if the circumstances of the polymerization are carefully controlled. Furthermore, it is inert in several chemical reactions. Consequently, along with other polymers,^17^ it is a suitable solid support for organocatalyst immobilization.

In a continuation of our previous work,^18^ in which we studied the effects of various modifications in the structure of cinchona organocatalysts on their catalytic activity, we aimed to create a PGMA solid carrier for cinchona squaramide organocatalysts containing a primary amino group at different positions. We immobilized these catalysts on the PGMA solid support by a covalent tether, and we investigated their catalytic activities in the Michael addition of pentane‐2,4‐dione to *trans*‐β‐nitrostyrene. Finally, the change in catalytic activity following their recovery was also studied.

## Results and Discussion

### Polymer synthesis

**Scheme 1:**
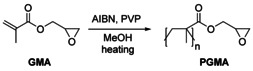
Preparation of PGMA by dispersion radical polymerization of PVP in the presence of AIBN initiator.

The PGMA polymer support was prepared by dispersion polymerization in methanol (Scheme 1). Our goal was to gain microspheres with narrow size distribution and high reactivity to allow easy chemical modification. We studied the effects of different factors on the polymerization and product quality (size, size distribution, morphology), including temperature, molar weight of the spherical stabilizer polyvinylpyrrolidone (PVP40, 40 kDa or PVP10, 10 kDa), and molar ratio of the components. As an initiator, azobisisobutyronitrile (AIBN) was used.

### Effect of temperature and composition on the polymerization

The effect of the temperature was examined at 50 °C, 60 °C, and at 65 °C using two different component ratios (Composition I and II, see Table S1 in the Supporting Information). At lower temperatures (50 °C and 60 °C), the microspheres formed agglomerates (Figure S1 in the Supporting Information). We only observed isolated spheres when the reaction was carried out at higher temperature (see Table S1 in the Supporting Information). More aggregates were found in reactions where Composition I was applied; however, the average diameter of the isolable microspheres was larger in this case.^19^

### Effect of the concentration of the spherical stabilizer

**Figure 1:**
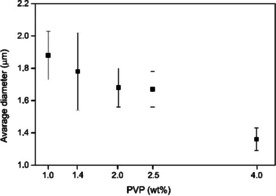
Size distribution of PGMA microspheres prepared with different PVP40 concentrations.

As the higher temperature has a beneficial influence on the polymerization, the effect of the molar ratio of PVP40 to the monomer (GMA) was investigated at 65 °C. We changed the amount of PVP40 between 1 and 4 wt % (compared with the reaction mixture, see Table S2 in the Supporting Information), and the average diameter of the microspheres increased when the amount of applied PVP40 was decreased (Figure 1).^20^ When larger microspheres were prepared, the size distribution widened, consequently, application of 4 wt % of PVP40 seemed ideal to gain a monodisperse product.

### Effect of molecular weight of spherical stabilizer

Horak and Shapoval compared the effect of PVPs with different molecular weights, and they observed that using PVP with smaller *M*_W_ resulted in larger microspheres of PGMA.16a The separation of larger particles by using centrifugation or filtration is easier, hence we also compared the effect of the PVPs with different molecular weights. However, the application of PVP10 resulted in larger and non‐spherical particles (Figure S2 in the Supporting Information), but with wide size distribution, thus the use of PVP40 is reasonable.

### Preparation and characterization of non‐cross‐linked PGMA microspheres

**Figure 2:**
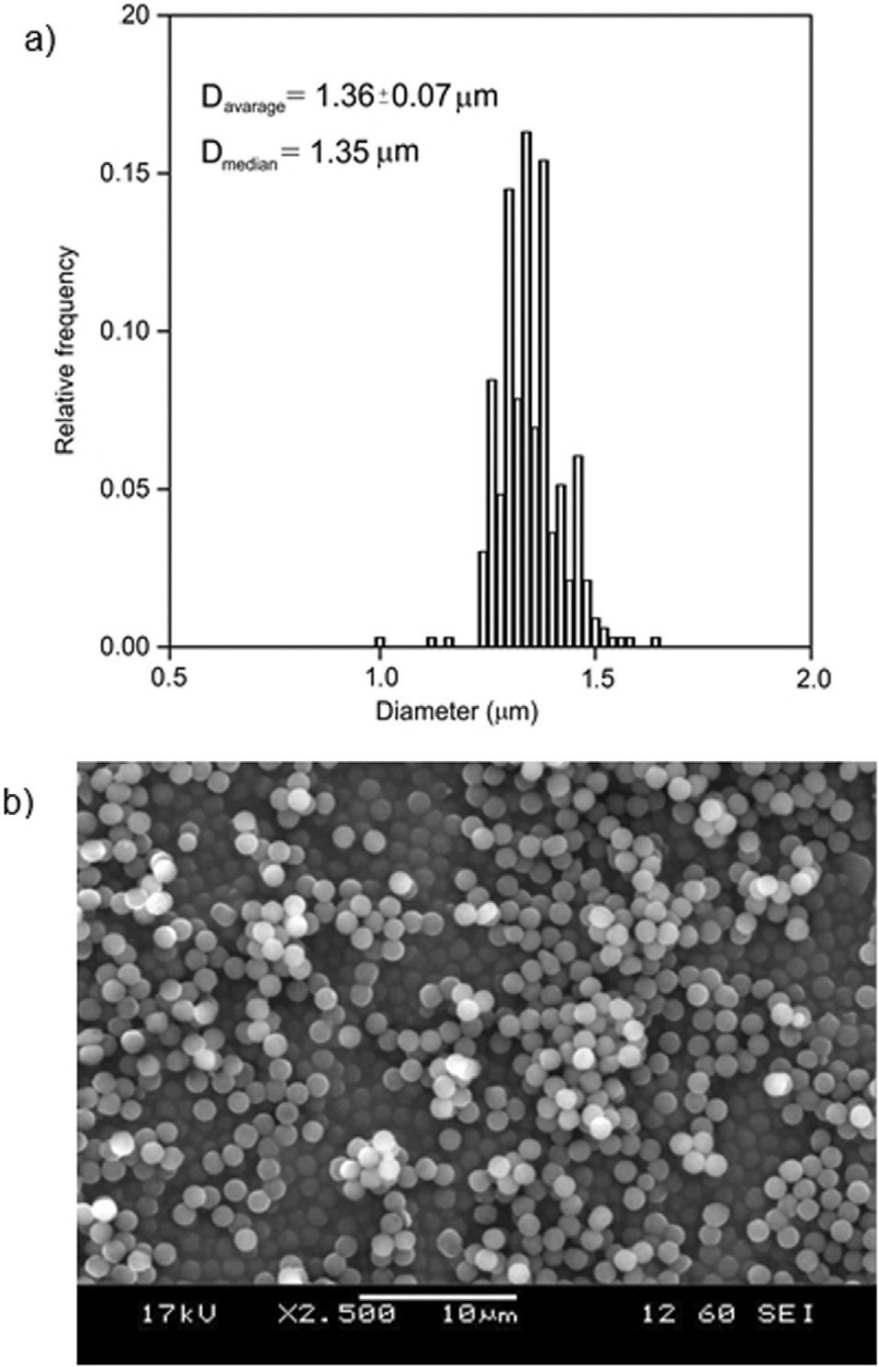
Size distribution (a) and SEM image of microspheres (b) prepared under the optimized conditions.

Following the study of polymerization parameters, non‐cross‐linked PGMA microspheres were prepared at 65 °C by using Composition II with PVP40. This method led to non‐porous monodisperse microspheres (Figure 2) with a narrow size distribution. In the case of a non‐porous catalyst carrier, the diffusion control is minimized during the catalytic reactions, however, the specific surface area of non‐porous materials is generally smaller.

### Subsequent cross‐linking

Catalyst supports are expected to be robust, inert, and insoluble during the application. Cross‐linked PGMA was found to be suitable as it is inert and insoluble in the frequently used solvents in asymmetric syntheses. Therefore, we used cross‐linking with ethylene glycol dimethacrylate (EGDMA) to further enhance the robustness of these support particles. EGDMA was applied between 15 and 35 wt % compared with the mass of the PGMA core. The subsequent cross‐linking was carried out at 60 °C in MeOH with AIBN as an initiator and PVP40 as a spherical stabilizer. This cross‐linking had no effect on the size distribution of the microspheres, furthermore, the formation of secondary spheres or aggregates was not observed.^19^

Testing the solubility at 25 °C, only the non‐cross‐linked PGMA was soluble in solvents such as toluene or DMF; moreover, swelling was also observed in other solvents. In comparison, the cross‐linked microspheres were not soluble in any solvent, and they showed swelling only in DMF. Consequently, this polymer is applicable for asymmetric Michael reactions in the preferred solvents such as EtOAc, CH_2_Cl_2_, or THF. In the case of any cross‐linked polymers, besides the solvents, in which the swelling/solubility measurements were taken, other components were not found in the fractions. This means that the cross‐linking is about 100 %, and it does not depend on the amount of the EGDMA in the region of 15 and 35 wt %.

Functional group analysis of the cross‐linked microspheres was performed by using the back‐titration method. Pyridinium chloride was added in excess, and then the remaining reagent was titrated with aqueous sodium hydroxide solution.^21^ On increasing the amount of EGDMA, the epoxy number decreased (Table 1). As it is a radical reaction, an excessive amount of EGDMA might reduce the number of accessible epoxy groups.^22^ As 15 wt % EGDMA already provided an insoluble polymer, it was not necessary to apply more cross‐linker. Thus, the number of accessible epoxy groups did not decrease considerably.

**Table 1:** The number of accessible epoxy groups in comparison to the amount of EGDMA applied during the cross‐linking of PGMA microspheres.

Entry	EGDMA [wt %]	Epoxy groups [mmol_epoxide_ g^−1^_polymer_]
1	0	6.38
2	15	5.87
3	20	5.23
4	25	5.13
5	30	4.69
6	35	4.25

### Catalyst synthesis

**Figure 3:**
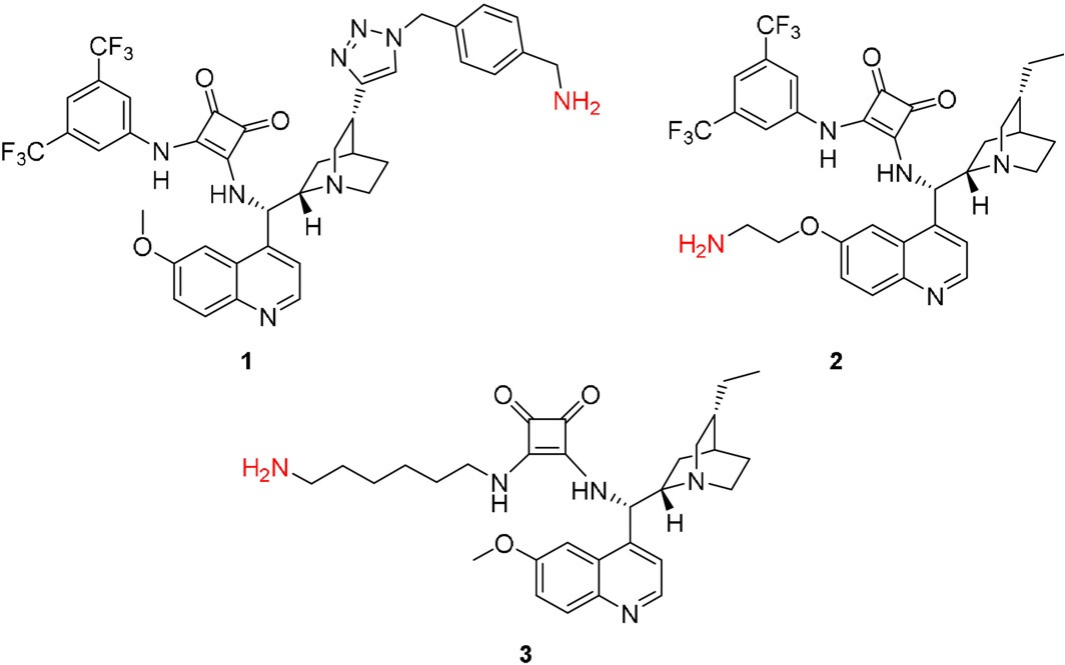
Schematics of cinchona squaramides modified with primary amino groups.

We chose cinchona squaramides to immobilize on the PGMA solid support, as they already have numerous successful applications in asymmetric transformations providing high yields and enantiomeric excess values.4c, 13, 18, 23 As epoxides readily react with primary amines,^24^ we synthesized three cinchona squaramides (**1**–**3**; Figure 3) with primary amino groups at different positions of the cinchona skeleton. The longer linker of **3** provided a bigger distance between the catalyst and the surface of the support.

**Scheme 2:**
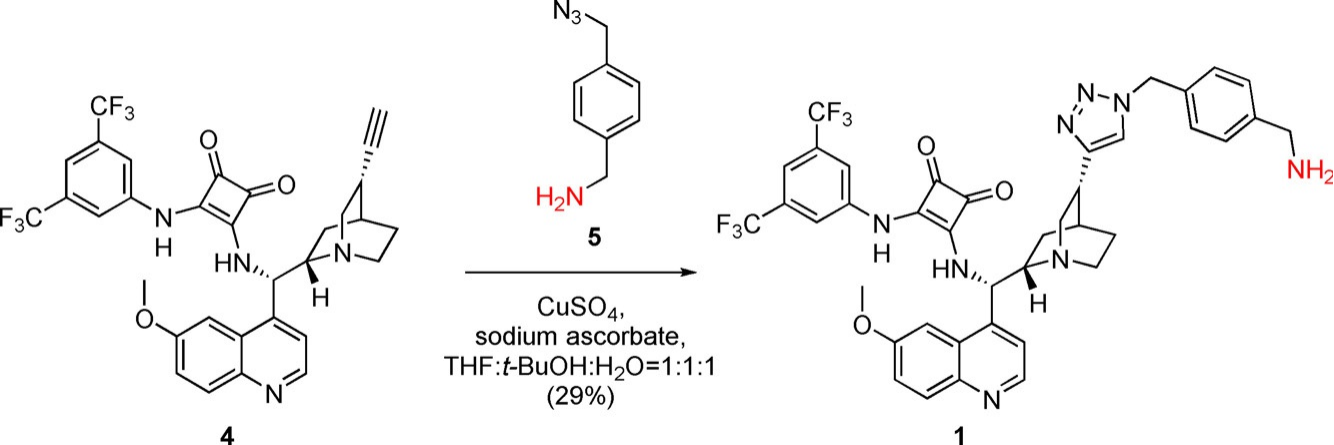
Synthesis of precatalyst **1** containing a rigid aromatic linker.

Cinchona squaramide modified with a rigid aromatic linker at the quinuclidine ring (**1**) was prepared in an azide–alkyne cycloaddition reaction of 1‐aminomethyl‐4‐azidomethylbenzene (**5**)^20^ and cinchona squaramide derivative containing an ethynyl group on the quinuclidine moiety (**4**, see Scheme 2).^18^

**Scheme 3:**
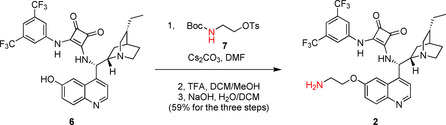
Synthesis of precatalyst **2** containing a short, flexible 2‐aminoethyl group as linker.

In the case of precatalyst **2**, a more flexible and shorter linker, namely a 2‐aminoethyl group, was incorporated into the cinchona skeleton at the quinoline unit. Demethylated cinchona squaramide (**6**)4c was reacted with *O*‐toluenesulfonyl‐*N*‐Boc‐ethanolamine (**7**). After the acidic removal of the protecting group of the intermediate, followed by neutralization, the corresponding primary amine **2** was obtained (Scheme 3).

**Scheme 4:**
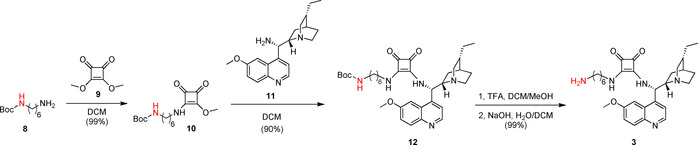
Synthesis of precatalyst with hexyl linker **3**.

Finally, precatalyst **3** was synthesized in a way that the squaramide unit was modified with a long, highly flexible C_6_ linker. In the reaction of commercially available *N*‐Boc‐1,6‐diaminohexane (**8**) and dimethyl squarate (**9**), half‐squaramide **10** was prepared. Next, this was reacted with cinchona amine^18^ **11** (Scheme 4). After deprotection of the intermediate **12**, we obtained the free amine containing a hexyl linker (**3**) with high yield (89 % for three steps).

**Scheme 5:**
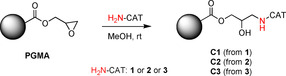
Preparation of the immobilized catalysts **C1**–**C3**.

As the last step of the synthesis, the immobilization of the primary amino group‐containing cinchona derivatives **1**, **2**, and **3** on cross‐linked PGMA (Scheme 5) was carried out in MeOH to gain three new solid‐supported organocatalysts (**C1**, **C2**, and **C3**). The amount of the immobilized precatalysts on the solid support was determined by elemental analysis of the catalysts (**C1**–**C3**) by using energy dispersive X‐ray analysis (EDX).

### Application of solid‐supported organocatalysts in the asymmetric Michael reaction, and their recycling by centrifugation

First, catalyst **C1** was applied in a reaction between pentane‐2,4‐dione (**14**) and *trans*‐β‐nitrostyrene (**13**) in two solvents (CH_2_Cl_2_ and EtOAc). The solvents were chosen based on how the non‐immobilized cinchona catalysts performed regarding the enantiomeric excess and yield reported in our previous work.^18^ Following the organocatalytic reaction (Table 2), the catalysts were recycled by centrifugation, and washed with the appropriate solvent. After the recovery of the catalyst, it was reused four times in the aforementioned reaction by using the same procedure.

**Table 2:** Test of catalyst **C1** in the Michael reaction using *trans*‐β‐nitrostyrene (**13**) and pentane‐2,4‐dione (**14**).^[a]^

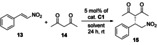			
Rounds	Solvent^[a]^	Yield [%]^[b]^	*ee* [%]^[c]^
1	EtOAc	97	6
2	EtOAc	98	6
3	EtOAc	98	5
4	EtOAc	98	4
5	EtOAc	98	4
1	CH_2_Cl_2_	87	29
2	CH_2_Cl_2_	89	31
3	CH_2_Cl_2_	90	21
4	CH_2_Cl_2_	90	21
5	CH_2_Cl_2_	90	17

[a] Reaction conditions: pentane‐2,4‐dione (**14**, 0.407 mmol) was added to the solution of *trans*‐β‐nitrostyrene (**13**, 0.157 mmol) in the presence of 5 mol % catalyst **C1** in 0.5 mL of solvent, then the resulting mixture was stirred at room temperature for 24 h. [b] Isolated yields. [c] Determined by chiral HPLC (the configuration of the major enantiomer is *S*).

Although catalyst **C1** provided Michael adducts with high yields, only small enantiomeric excess values were observed. Also, although the yields remained unchanged, a small decrease in the *ee* can be seen. To confirm that the low enantiomeric excess values are not caused by the PGMA support acting as a competitive catalyst, the Michael reaction was carried out using non‐modified PGMA (Table 3). In this procedure, product formation was not observed, therefore, the solid support in itself does not catalyze this reaction. Homogeneous cinchona‐based organocatalysts usually give higher *ee* values in CH_2_Cl_2_,^18^ therefore, the Michael reaction was carried out with the non‐immobilized (homogeneous) precatalysts as well (Table 3). As the *ee* was significantly higher when using CH_2_Cl_2_, catalysts **C2** and **C3** were tested only in this solvent. Following the first round, they were also recycled four times by centrifugation (Table 3). After five cycles, degradation or deformation of any cinchona‐modified PGMA catalyst was not observed based on the SEM images (Figure S3 in the Supporting Information). When catalyst **C2** or **C3** was applied, the enantiomeric excess values were higher (up to 79 %) than in the case of **C1**. Among the immobilized catalysts, the best results were obtained with catalyst **C2**.

**Table 3:** Test of catalysts **C2** and **C3** in the Michael reaction using *trans*‐β‐nitrostyrene (**13**) and pentane‐2,4‐dione (**14**).^[a]^

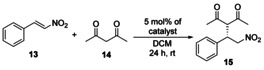			
Rounds	Catalyst	Yield [%]^[b]^	*ee* [%]^[c]^
–	non‐modified PGMA^[d]^	not observed	–
–	**1**	91	81
–	**2**	92	92
–	**3**	87	85
1	**C2**	89	78
2	**C2**	87	79
3	**C2**	82	75
4	**C2**	80	73
5	**C2**	78	74
1	**C3**	87	59
2	**C3**	87	58
3	**C3**	82	56
4	**C3**	83	56
5	**C3**	79	53

[a] Reaction conditions: pentane‐2,4‐dione (**14**, 0.407 mmol) was added to the solution of *trans*‐β‐nitrostyrene (**13**, 0.157 mmol) in the presence of 5 mol % catalyst **1**, **2**, **3**, **C2**, or **C3** in 0.5 mL of CH_2_Cl_2_, then the resulting mixture was stirred at room temperature for 24 h. [b] Isolated yields. [c] Determined by chiral HPLC (the configuration of the major enantiomer is *S*). [d] 50 mg of non‐modified, cross‐linked PGMA was used instead of catalysts under the same reaction conditions.

The better results can be explained by mechanistic reasons.^25^ This Michael addition reaction begins with deprotonation of the dioxo compound (forming a nucleophile), and in the case of these catalysts, the deprotonation depends on the basicity of the tertiary amine. In the case of **C1**, the substituent at the quinuclidine motif is a 1,2,3‐triazole‐4‐yl unit, in contrast to **C2** and **C3** where an ethyl group is connected to the ring in this position. Therefore, the difference in the yields might be caused by the electronic effects of these substituents.

Regarding the *ee*, **C3** contains a linker with a longer chain, but the squaramide NH groups are more acidic when they are bound to an electron‐withdrawing group. Consequently, stronger hydrogen bonds could form between the corresponding substrate and the **C2** catalyst containing a bis(trifluoromethyl)phenyl modified squaramide moiety. This can result in stronger interaction between the catalyst and substrates, allowing a more definite stereocontrol of the reaction.23e, 23f, 25, 26

However, precatalysts **1**–**3** contain primary amino groups that compete with the basic nitrogen of the quinuclidine moiety, the selectivities given by these precatalysts were higher than those that resulted when the corresponding immobilized ones were applied (**C1**–**C3**). This can be explained by the commonly experienced lower productivity and insufficient selectivity of the heterogeneous catalyst compared with homogeneous ones.

As the best results were obtained by the application of recyclable catalyst **C2** at room temperature (and the enantioselectivity may increase by decreasing the temperature), we also performed the Michael reaction at 0 °C using catalyst **C2** (Table 4).

**Table 4:** Test of catalyst **C2** in the Michael reaction using *trans*‐β‐nitrostyrene (**13**) and pentane‐2,4‐dione (**14**)^[a]^ at lower temperature.

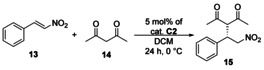		
Rounds	Yield [%]^[b]^	*ee* [%]^[c]^
1	84	96
2	80	96
3	80	96
4	75	96
5	76	96

[a] Reaction conditions: pentane‐2,4‐dione (**14**, 0.407 mmol) was added to the solution of *trans*‐β‐nitrostyrene (**13**, 0.157 mmol) in the presence of 5 mol % catalyst **C2** in 0.5 mL of CH_2_Cl_2_, then the resulting mixture was stirred at room temperature for 24 h. [b] Isolated yields. [c] Determined by chiral HPLC (the configuration of the major enantiomer is *S*).

**Figure 4:**
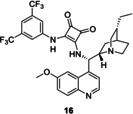
Schematic of the cinchona squaramide used as a reference catalyst.

As our results in Table 4 show, comparing them to that obtained at room temperature, the enantioselectivity is higher when the reaction is run at 0 °C. After five runs, the enantioselectivity did not change, but a slight decrease in yields was observed. As control experiments, we performed this Michael reaction by using the non‐immobilized precatalyst **2** and a non‐modified cinchona squaramide (**16**, Figure 4)^18^ in the presence of the polymer support (PGMA).

At room temperature, the presence of PGMA did not affect the yield, but under these conditions, the non‐immobilized catalyst **2** resulted in a slightly lower *ee* (86 %) compared with that without PGMA (92 %; Table 3 and Table 5). Moreover, the mixture of PGMA and cinchona squaramide **16** gave a similar yield (97 %) and enantioselectivity (88 %) as using only catalyst **16**, which is described in the literature (99 % yield, 85 % *ee*) in the Michael reaction between *trans*‐β‐nitrostyrene (**13**) and pentane‐2,4‐dione (**14**).^18^ Therefore, it can be stated that the presence of non‐modified PGMA had no effect on the catalytic activity of cinchona catalysts in the studied asymmetric Michael reactions.

**Table 5:** Test of the mixture of PGMA and homogeneous cinchona catalysts **C2** or **16** in the Michael reaction using *trans*‐β‐nitrostyrene (**13**) and pentane‐2,4‐dione (**14**).^[a]^

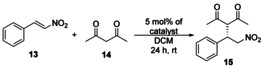		
Catalyst	Yield [%]^[b]^	*ee* [%]^[c]^
**2**+PGMA	90	86
**16**+PGMA	97	88

[a] Reaction conditions: 50 mg of non‐modified, cross‐linked PGMA and pentane‐2,4‐dione (**14**, 0.407 mmol) were added to the solution of *trans*‐β‐nitrostyrene (**13**, 0.157 mmol) in the presence of 5 mol % catalyst **2** or **16** in 0.5 mL of CH_2_Cl_2_, then the resulting mixture was stirred at room temperature for 24 h. [b] Isolated yields. [c] Determined by chiral HPLC (the configuration of the major enantiomer is *S*).

## Conclusion

We have prepared three new precatalysts (**1**–**3**) by modifying cinchona squaramide organocatalysts. These derivatizations were made at three different positions of these bifunctional catalysts: one linker was connected to the catalyst at the quinuclidine ring (**1**), another at the quinoline moiety (**2**), and the last one at the squaramide unit (**3**). These linkers contained a terminal primary amino group; hence, these catalysts were easily immobilized on PGMA microspherical polymer containing epoxy groups. To the best of our knowledge, this is the first utilization of PGMA as a solid support for organocatalysts.

The prepared three new solid‐supported catalysts **C1**–**C3** were applied in a Michael addition reaction by using *trans*‐β‐nitrostyrene (**13**) and pentane‐2,4‐dione (**14**) with high preparative yields (up to 98 %) and enantioselectivities (up to 96 % *ee*). Finally, these polymer‐grafted catalysts were easily recovered by centrifugation and applied in five consecutive runs with only a slight decrease in yields and enantioselectivities. Our results show that immobilization of cinchona squaramide organocatalyst on cross‐linked PGMA solid support is a feasible method to resolve the inconvenient recycling of the corresponding homogeneous organocatalyst. Finally, the modification of PGMA microspheres with amino functionalized organocatalysts is a generally applicable approach that could be utilized for a wide variety of organocatalysts.

## Experimental Section

### General

Infrared spectra were recorded with a Bruker Alpha‐T FTIR spectrometer. Optical rotations were measured with a PerkinElmer 241 polarimeter that was calibrated by measuring the optical rotations of both enantiomers of menthol. NMR spectra were taken at the Directorate of Drug Substance Development, Egis Pharmaceuticals PLC., with a Bruker Avance III HD (at 600 MHz for ^1^H and at 150 MHz for ^13^C spectra) or at the Department of Inorganic & Analytical Chemistry, Budapest University of Technology and Economics, with a Bruker DRX‐500 Avance spectrometer (at 500 MHz for ^1^H and at 125 MHz for ^13^C spectra). The exact mass measurements were performed by using a Q‐TOF Premier mass spectrometer (Waters Corporation, 34 Maple St, Milford, MA, USA) in positive electrospray ionization mode. Solvents were purchased from Merck (Darmstadt, Germany) unless stated otherwise. Starting materials and reagents were purchased from Sigma–Aldrich (Saint Louis, MO, USA), unless otherwise stated, and used without purification. *Tert‐*butyl(6‐aminohexyl)carbamate was purchased from SIA Enamine Ltd. (Riga, Latvia). The enantiomeric excess (*ee*) values were determined by chiral HPLC with a PerkinElmer Series 200 instrument equipped with Phenomenex Lux^®^ 5 μm Cellulose‐1 column (250×4.6 mm ID), an 85:15 mixture of hexane/ethanol was used as the eluent with a flow rate of 0.8 mL min^−1^. The column temperature was 20 °C. UV detector *λ*=254 nm. Melting points were taken with a Boetius micro‐melting point apparatus, and they were uncorrected. Silica gel 60 F_254_ (Merck) plates were used for TLC. The spots of materials on TLC plates were visualized by UV light at 254 nm. Silica gel 60 (70–230 mesh, Merck) was used for column chromatography. Ratios of solvents for the eluents are given in milliliters. All PGMA nanoparticles were analyzed by using a JEOL JSM‐5500LV scanning electron microscope (SEM) at high vacuum with the corresponding accelerating voltage. For better imaging, samples were coated with a gold nanofilm layer by using a vacuum nebulizer. Solubility and swelling test: 1.00 g of the PGMA was stirred in 100 mL of the following solvents: MTBE (methyl *tert*‐butyl ether), EtOAc, DMF, CH_2_Cl_2_, MeCN. After 24 h, it was centrifuged, and SEM images were taken of each sample; soluble fractions were analyzed by HPLC. Elemental analyses were performed in the Microanalytical Laboratory of the Department of Organic Chemistry, Institute for Chemistry, L. Eötvös Loránd University, Budapest, Hungary. The elemental analysis of the PGMA and catalyst **C1**–**C3** samples without gold nanolayer was carried out with energy dispersive X‐ray analysis (EDX with Si(Li) detector) applying 15 kV accelerating voltage and sampling time of 40 s.

### Synthesis of solid supports, precatalysts, and immobilized catalysts

#### General procedure for the preparation of non‐cross‐linked PGMA microspheres (Composition II)

PVP (2 g) was dissolved in MeOH (50 mL) in a 100 mL two‐necked round‐bottomed flask set up with an inner thermometer. GMA (5 g) was added to this mixture. Then, a solution of AIBN (50 mg) in MeOH (4 mL) was added to the reaction mixture. While the components were added to the solution, the mixture was continuously purged with nitrogen. Then, the reaction mixture was capped and sealed. It was stirred with a magnetic stirrer at the temperatures given in Tables S1 and S2 (in the Supporting Information). After 48 h, the mixture was cooled down to room temperature, then transferred into centrifuge tubes and was centrifuged for 8 min at 5000 rpm. The mother liquor was exchanged with pure MeOH, and the product was suspended by using an ultrasonic bath, then it was centrifuged. This washing was repeated three times. Finally, the product was washed with distilled water, and it was allowed to dry at room temperature in a Petri dish. For yields, see Tables S1 and S2 (in the Supporting Information).

GMA was treated with aqueous NaOH solution (30 wt %) right before its use to remove the inhibitor monomethyl ether hydroquinone. To GMA (10 mL), an aqueous NaOH solution (30 wt %, 1 mL) was added and stirred vigorously for 15 min. The phases were separated, then the organic phase was washed with water (5 mL). After separating the phases, GMA was dried over MgSO_4_. After filtering the drying agent, GMA was ready to use.

#### General procedure for the preparation of cross‐linked PGMA microspheres (Composition VII, see Table S3 in the Supporting Information)

In a 100 mL two‐necked round‐bottomed flask equipped with an inner thermometer, the non‐cross‐linked PGMA (2 g) was suspended in MeOH (30 mL) by using an ultrasonic bath. A solution of PVP (2 g) in MeOH (20 mL) and a solution of EGDMA (300 mg) in MeOH (4 mL) were added to the suspension of PGMA. Then, a solution of AIBN (50 mg) in MeOH (4 mL) was added to the reaction mixture. While the components were added to the solution, the mixture was continuously purged with nitrogen. Then, the reaction mixture was capped and sealed. It was stirred with a magnetic stirrer for 24 h at 60 °C. After 24 h, the mixture was cooled down to room temperature, then transferred into centrifuge tubes and was centrifuged for 8 min at 5000 rpm. The mother liquor was exchanged with pure MeOH, and the product was suspended by using an ultrasonic bath, then centrifuged. This washing was repeated three times. Finally, the product was washed with distilled water, and it was allowed to dry at room temperature in a Petri dish. For yields, see Table S3 (in the Supporting Information).

EGDMA was treated by aqueous NaOH solution (30 wt %) right before its use to remove the inhibitor monomethyl ether hydroquinone. To EGDMA (2 mL), an aqueous NaOH solution (30 wt %, 0.2 mL) was added and stirred vigorously for 15 min. The phases were separated, then the organic phase was washed with water (2 mL). After separating the phases, EGDMA was dried over MgSO_4_. After filtering the drying agent, EGDMA was ready to use.

**3‐(((*S*)‐((1*S*,2*S*,4*S*,5*R*)‐(−)‐5‐(1‐(4‐(Aminomethyl)benzyl)‐1** ***H*** **‐1,2,3‐triazol‐4‐yl)quinuclidine‐2‐yl)(6‐methoxyquinolin‐4‐yl)methyl)amino)‐4‐((3,5‐bis(trifluoromethyl)phenyl)amino)cyclobut‐3‐ene‐1,2‐dione (1)**: (4‐(Azidomethyl)phenyl)methanamine (**5**, 63.2 mg, 0.389 mmol) was dissolved in a mixture of THF (0.32 mL) and water (0.16 mL). Sodium ascorbate (5.9 mg, 0.030 mmol) and copper sulfate pentahydrate (3.7 mg, 0.015 mmol) were added to this solution. Water (0.16 mL) was added to a solution of ethynyl cinchona squaramide (**4**, 188.3 mg, 0.300 mmol) in THF (0.32 mL), and this solution was added dropwise to the solution of (4‐(azidomethyl)phenyl)methanamine (**5**), and it was stirred at room temperature. After 48 h, when the cinchona squaramide was consumed, the solvent was evaporated under reduced pressure. The crude product was purified by preparative TLC on silica gel by using a mixture of CH_2_Cl_2_/methanol/25 % NH_4_OH (10:1:0.01) as an eluent. The product is a yellow solid (78 mg, 29 %). TLC (SiO_2_ TLC; CH_2_Cl_2_/methanol/25 % NH_4_OH=10:1:0.01, *R*_f_=0.26); m.p.: 233–235 °C; [*α*]20D =−42.0 (*c*=1.00, acetone); IR: *ν*_max_=3401, 3247, 3084, 3045, 2963, 2880, 2349, 1796, 1679, 1624, 1607, 1559, 1514, 1444, 1380, 1331, 1280, 1183, 1133, 1023 cm^−1^; ^1^H NMR (500 MHz, acetone‐d_6_): *δ*=8.61 (1 H, s), 8.04 (2 H, s), 7.89 (1 H, d, *J*_H,H_=9.0 Hz), 7.83 (1 H, s), 7.80 (1 H, s), 7.49 (1 H, overlapped), 7.44 (1 H, overlapped), 7.30 (1 H, d, *J*_H,H_=9.0 Hz), 7. 08 and 7.16 (2×2 H, AA′BB′, *J*_AB_=7.0 Hz, Ph‐H), 6.23 (1 H, broad), 5.47 (1 H, s), 3.96 (3 H, s), 3.82 (1 H, m), 3.55 (2 H, m, overlapped), 3.50 (3 H, m, overlapped), 3.39 (2 H, m), 3.29 (1 H, s), 3.03 (2 H, m), 2.78 (1 H, m), 1.85 (1 H, m), 1.63 (1 H, m), 1.43 (1 H, m), 1.27 (1 H, m), 0.72 ppm (1 H, m); ^13^C NMR (125 MHz, acetone‐d_6_): *δ*=184.3, 177.3, 170.8, 163.6, 159.7 (q, *J*_C,F_=26 Hz), 158.2, 148.8, 138.5, 129.5 (q, *J*_C,F_=28 Hz), 128.1, 127.4, 127.3, 123.3, 109.6, 94.6, 75.1, 72.1, 65.9, 61.7, 59.4, 57.2, 55.0, 38.3, 37.1, 35.1, 27.4, 26.8, 25.0, 22.5, 18.1, 13.2 ppm; HRMS‐ESI+ (*m*/*z*): [*M*−H^+^] calcd for C_40_H_35_F_6_N_8_O_3_: 789.2742; found: 789.2746; elemental analysis calcd (%): C 60.76, H 4.59, F 14.41, N 14.17; found: C 60.71, H 4.64, F 14.40, N 14.16.

To the best of our knowledge, the synthesis of **1** has not been reported so far.


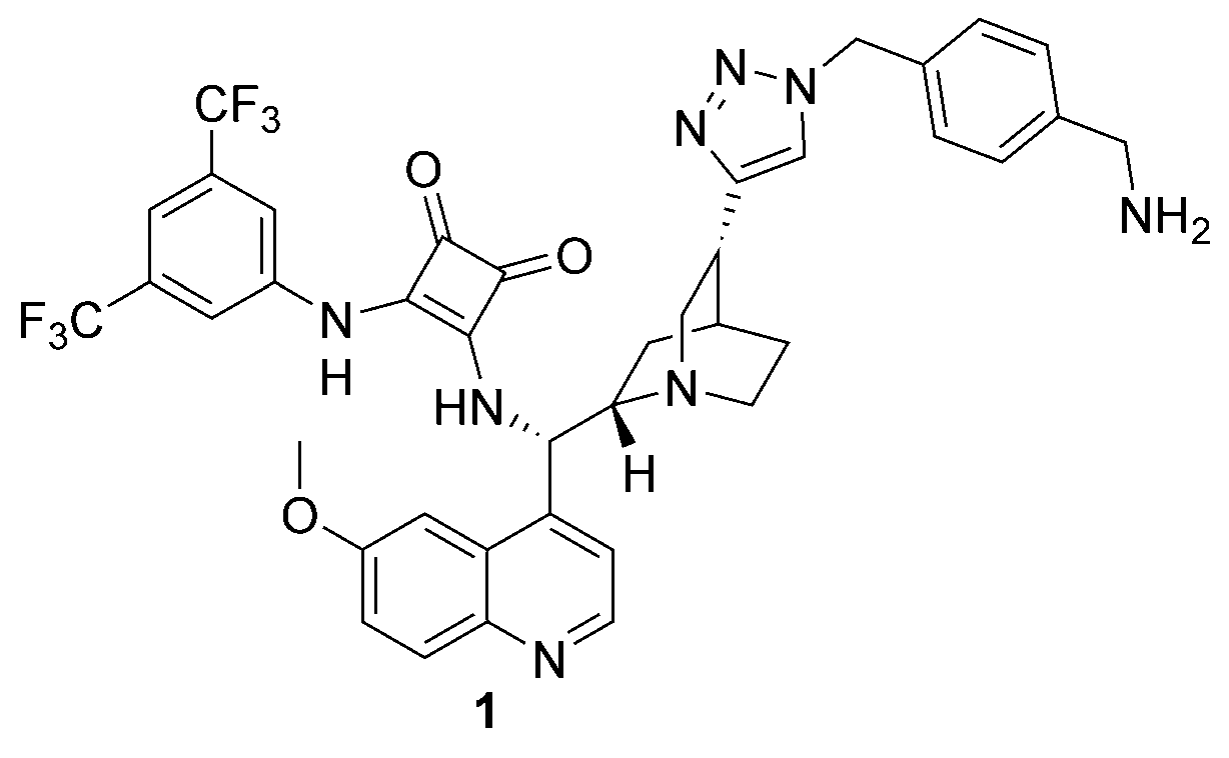


**3‐(((*S*)‐(−)‐(6‐(2‐Aminoethoxy)quinolin‐4‐yl)((1*S*,2*S*,4*S*,5*R*)‐5‐ethylquinuclidin‐2‐yl)methyl)amino)‐4‐((3,5‐bis(trifluoromethyl)phenyl)amino)cyclobut‐3‐ene‐1,2‐dione (2)**: Cesium carbonate (870 mg, 2.67 mmol) was added to a solution of cinchona squaramide containing a phenolic hydroxyl group at the quinoline moiety (**6**, 660 mg, 1.07 mmol) in DMF (16 mL) and this mixture was stirred for 15 min. To this mixture, tosylate **7** (404 mg, 1.28 mmol) was added dissolved in DMF (3 mL), and the solution was stirred for 2 days at room temperature. Then, the solvent was evaporated under reduced pressure. The residue was suspended in CH_2_Cl_2_ (20 mL), filtered, and the solvent was evaporated under reduced pressure. This crude product was dissolved in CH_2_Cl_2_ (12 mL), and TFA (5.51 g, 3.7 mL, 48.4 mmol) was added to this solution and stirred for 1 h at room temperature. Then, the solution was cooled to 0 °C, and the amine was liberated from its salt by aqueous NaOH solution (10 wt %). This mixture was extracted with CH_2_Cl_2_ (3×20 mL), dried over MgSO_4_, and filtered. Finally, the solvent was evaporated under reduced pressure. The product is a yellow solid (413 mg, 99 %). TLC (SiO_2_ TLC; CH_2_Cl_2_/methanol/25 % NH_4_OH=3:1:0.01, *R*_f_=0.28); m.p.: 196–199 °C; [*α*]20D =−62.5 (*c*=1.00, MeOH); IR: *ν*_max_=3431, 3076, 2960, 2851, 2378, 1949, 1724, 1622, 1592, 1578, 1507, 1455, 1438, 1409, 1371, 1340, 1263, 1243, 1169, 1099, 1043, 1018 cm^−1^; ^1^H NMR (600 MHz, DMSO‐d_6_): *δ*=8.83 (1 H, d, *J*_H,H_=4.8 Hz), 8.11 (2 H, s), 8.04 (1 H, d, *J*_H,H_=9.0 Hz), 7.82 (1 H, s), 7.72 (1 H, d, *J*_H,H_=4.8 Hz), 7.63 (1 H, s), 7.50 (1 H, dd, *J*_1,H,H_=3.0 Hz, *J*_2,H,H_=9.0 Hz), 6.08 (1 H, broad), 4.41 (1 H, m), 4.37 (1 H, m), 3.36 (3 H, m), 3.26 (2 H, m), 3.14 (2 H, m), 2.63 (1 H, m), 2.43 (1 H, m), 1.55 (1 H, overlapped), 1.52 (1 H, overlapped), 1.39 (3 H, m), 1.33 (3 H, m), 1.11 (1 H, s), 0.80 (3 H, t, *J*_H,H_=7.2 Hz), 0.55 ppm (1 H, broad); ^13^C NMR (150 MHz, DMSO‐d_6_): *δ*=185.0, 180.0, 168.8, 163.3, 158.6 (q, *J*_C,F_=31 Hz), 156.7, 148.4, 144.5, 143.7, 141.5, 131.9, 131.4 (q, *J*_C,F_=33 Hz), 127.5, 126.1, 124.3, 122.5, 120.6, 120.3, 119.8, 118.5, 118.3, 116.3, 115.0, 114.4, 102.2, 67.2, 65.4, 64.3, 59.2, 57.2, 55.1, 53.2, 40.4, 38.8, 36.9, 31.5, 28.2, 27.1, 25.9, 25.2, 12.1 ppm; HRMS‐ESI+ (*m*/*z*): [*M*+H^+^] calcd for C_33_H_33_F_6_N_5_O_3_: 662.2566; found: 662.2569; elemental analysis calcd (%): C 59.91, H 5.03, F 17.23, N 10.58; found: C 59.87, H 5.07, F 17.23, N 10.56.


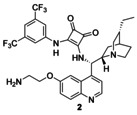


To the best of our knowledge, the synthesis of **2** has not been reported so far.

**3‐((6‐Aminohexyl)amino)‐4‐(((*S*)‐((1*S*,2*S*,4*S*,5*R*)‐(−)‐5‐ethyl quinuclidin‐2‐yl)(6‐methoxyquinolin‐4‐yl)methyl)amino) cyclobut‐3‐ene‐1,2‐dione (3)**: The *N*‐Boc protected squaramide (**12**, 1.52 g, 2.46 mmol) was dissolved in CH_2_Cl_2_ (30 mL), and TFA (11.0 g, 7.4 mL, 96.8 mmol) was added to this solution and stirred for 1 h at room temperature. Then, the solution was cooled to 0 °C, and the amine was liberated from its salt by using an aqueous NaOH solution (10 wt %). This mixture was extracted with CH_2_Cl_2_ (3×20 mL), dried over MgSO_4_, and filtered. The solvent was evaporated under reduced pressure, and the crude product was purified by column chromatography on silica gel using a mixture of CH_2_Cl_2_/methanol/25 % NH_4_OH=3:1:0.01 as an eluent. The product is a white crystalline solid (1.27 g, 99 %). TLC (SiO_2_ TLC; CH_2_Cl_2_/methanol/25 % NH_4_OH=3:1:0.01, *R*_f_=0.4); m.p.: 133–135 °C; [*α*]20D =−64.8 (*c*=1.00, chloroform); IR: *ν*_max_=3430, 3239, 3154, 3077, 2962, 2919, 2851, 2645, 2553, 2142, 2052, 1934, 1724, 1595, 1559, 1519, 1490, 1473, 1449, 1410, 1387, 1340, 1313, 1262, 1231, 1210, 1170, 1097, 1029, 1018 cm^−1^; ^1^H NMR (500 MHz, CDCl_3_): *δ*=8.77 (1 H, s), 8.03 (1 H, d, *J*_H,H_=9.0 Hz), 7.85 (1 H, s), 7.65 (1 H, s), 7.40 (1 H, d, *J*_H,H_=9.0 Hz), 6.26 (1 H, s), 5.73 (2 H, broad), 4.02 (3 H, s), 3.53 (2 H, m), 3.38 (2 H, overlapped), 3.23 (1 H, overlapped), 2.81 (3 H, m, overlapped), 2.51 (1 H, m), 1.02–1.70 (15 H, m, overlapped), 0.79 ppm (4 H, m); ^13^C NMR (125 MHz, CDCl_3_): *δ*=182.8, 182.0, 179.1, 169.9, 168.2, 167.2, 158.7, 158.6, 147.7, 144.8, 131.7, 127.8, 122.6, 101.3, 60.0, 57.1, 56.1, 53.4, 46.0, 44.0, 40.8, 39.9, 36.6, 36.4, 34.5, 30.3, 29.7, 28.5, 27.5, 27.1, 27.0, 25.7, 25.5, 25.3, 25.0, 24.7, 23.3, 14.8, 11.9, 11.8, 10.8, 8.4 ppm; HRMS‐ESI+ (*m*/*z*): [*M*+H^+^] calcd for C_30_H_41_N_5_O_3_: 520.3288; found: 520.3290; elemental analysis calcd (%): C 69.34, H 7.95, N 13.48; found: C 69.30, H 7.98, N 13.48.


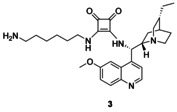


To the best of our knowledge, the synthesis of **3** has not been reported so far.

***tert‐*** **Butyl(6‐((2‐methoxy‐3,4‐dioxocyclobut‐1‐en‐1‐yl)amino)hexyl)carbamate (10)**: *tert‐*Butyl(6‐aminohexyl)carbamate (**8**, 761 mg, 3.52 mmol) was dissolved in CH_2_Cl_2_ (3 mL), and it was added dropwise to a solution of dimethyl squarate (**9**, 500 mg, 3.52 mmol) in CH_2_Cl_2_ (3 mL). After 12 h, the reaction was complete, and the solvent was evaporated under reduced pressure. The crude product was purified by column chromatography on silica gel using a mixture of CH_2_Cl_2_/methanol (20:1) as an eluent. The product is a white crystalline solid (1.15 g, 99 %). TLC (SiO_2_ TLC; CH_2_Cl_2_/methanol=20:1 *R*_f_=0.28); m.p.: 74–76 °C; IR: *ν*_max_=2930, 2870, 1722, 1690, 1618, 1581, 1504, 1470, 1454, 1430, 1354, 1243, 1221, 1099, 1075, 1027, 1005 cm^−1^; ^1^H NMR (500 MHz, CDCl_3_): *δ*=6.85 (1 H, broad), 4.59 (1 H, broad), 4.41 (3 H, overlapped), 3.65 (1 H, broad), 3.42 (1 H, m), 3.11 (2 H, m), 1.62 (2 H, m), 1.48 (2 H, m), 1.44 (9 H, s), 1.36 ppm (4 H, m); ^13^C NMR (125 MHz, CDCl_3_): *δ*=189.6, 182.9, 177.6, 172.2, 100.0, 79.2, 60.5, 44.7, 34.5, 30.4, 30.0, 28.4, 26.1, 25.8 ppm; MS‐ESI+ (*m*/*z*): [*M*+H^+^] calcd for C_16_H_26_N_2_O_5_: 327.19; found: 327.20; elemental analysis calcd (%): C 58.88, H 8.03, N 8.58; found: C 58.83, H 8.08, N 8.56.


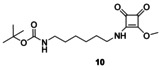


To the best of our knowledge, the synthesis of **10** has not been reported so far.

***tert‐*** **Butyl(6‐((2‐(((*S*)‐((1*S*,2*S*,4*S*,5*R*)‐(−)‐5‐ethylquinuclidin‐2‐yl)(6‐methoxyquinolin‐4‐yl)methyl)amino)‐3,4‐dioxocyclobut‐1‐en‐1‐yl)amino)hexyl)carbamate (12)**: Cinchona amine^18^ (**11**, 800 mg, 2.46 mmol) was added to a solution of half squaramide (**10**, 803 mg, 2.46 mmol) in CH_2_Cl_2_ (6 mL). After stirring overnight, the reaction was completed. The solvent was evaporated under reduced pressure, and the crude product was purified by column chromatography on silica gel by using a mixture of CH_2_Cl_2_/methanol/25 % NH_4_OH=10:1:0.01 as an eluent. The product is a white crystalline solid (1.37 g, 90 %). TLC (SiO_2_ TLC; CH_2_Cl_2_/methanol/25 % NH_4_OH=10:1:0.01, *R*_f_=0.6); m.p.: 131–132 °C; [*α*]20D =−78.1 (*c*=1.00, chloroform); IR: *ν*_max_=3104, 2961, 2876, 1722, 1663, 1619, 1573, 1511, 1467, 1434, 1379, 1362, 1252, 1230, 1197, 1161, 1117, 1099, 1064, 1019, 1003 cm^−1^; ^1^H NMR (500 MHz, CDCl_3_): *δ*=8.81 (1 H, s), 8.07 (1 H, d, *J*_H,H_=9.5 Hz), 7.80 (1 H, s), 7.68 (1 H, s), 7.45 (1 H, d, *J*_H,H_=9.0 Hz), 6.36 (1 H, broad), 5.32 (1 H, s), 4.79 (1 H, broad), 4.12 (1 H, overlapped), 4.04 (3 H, s), 3.98 (1 H, overlapped), 3.48 (3 H, m), 3.14 (1 H, overlapped), 3.04 (2 H, overlapped), 2.95 (1 H, overlapped), 2.00 (1 H, m), 1.92 (3 H, m, overlapped), 1.73 (1 H, m), 1.51 (3 H, overlapped), 1.44 (9 H, s), 1.37 (4 H, overlapped), 1.23 (3 H, m), 1.04 (1 H, m), 0.90 ppm (3 H, m); ^13^C NMR (125 MHz, CDCl_3_): *δ*=186.7, 183.0, 179.2, 170.8, 166.3, 159.1, 156.20, 146.5, 144.9, 132.0, 127.6, 123.0, 119.0, 99.9, 56.3, 53.4, 44.4, 40.3, 32.1, 29.7, 28.5, 28.0, 26.7, 26.1, 25.8, 25.3, 24.51, 24.49, 23.1, 14.3, 13.3, 11.6, 8.5 ppm; MS‐ESI+ (*m*/*z*): [*M*+H^+^] calcd for C_35_H_49_N_5_O_5_: 620.3812; found: 620.3810; elemental analysis calcd (%): C 67.82, H 7.97, N 11.30; found: C 67.78, H 8.00, N 11.29.


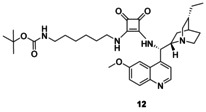


To the best of our knowledge, the synthesis of **12** has not been reported so far.

#### General procedure for the immobilization of precatalysts

Cross‐linked PGMA (80 mg) was suspended in MeOH (0.5 mL) by using an ultrasonic bath. Precatalyst (80 mg, **1**, **2**, or **3**) was added to this suspension, and this mixture was stirred for 48 h at room temperature. Then, the mixture was transferred into centrifuge tubes and was centrifuged for 8 min at 8000 rpm. The mother liquor was exchanged with pure MeOH, and the product triturated with the supernatant by using an ultrasonic bath, then it was centrifuged. This washing was repeated three times. Finally, the product was washed with distilled water, and it was allowed to dry at room temperature. The catalyst content of the final product was determined by elemental analysis by using energy dispersive X‐ray analysis (EDX):

**C1**: 0.0388 mmol_precatalyst_ g^−1^_immobilized catalyst_;

**C2**: 0.183 mmol_precatalyst_ g^−1^_immobilized catalyst_;

**C3**: 0.193 mmol_precatalyst_ g^−1^_immobilized catalyst_.

Application of immobilized catalysts in asymmetric Michael addition

#### General procedure for Michael addition of pentane‐2,4‐dione (14) to trans‐β‐nitrostyrene (13)

To a solution of *trans*‐β‐nitrostyrene (**13**, 15 mg, 0.1 mmol) in the solvent (0.5 mL) shown in Tables 3–4, catalyst **C1**, **C2**, or **C3** was added. Then, pentane‐2,4‐dione (**14**, 20 μL, 19.6 mg, 0.20 mmol) was added to this solution, and the resulting mixture was stirred at the temperature shown in Tables 3–4. After 24 h, the reaction mixture was transferred into a small centrifuge tube and was centrifuged for 8 min at 8000 rpm. The mother liquor was exchanged with pure solvent (0.5 mL), then the catalyst was suspended by using an ultrasonic bath, and finally, it was centrifuged. This washing was repeated three times. After washing, the volatile components from the combined mother liquors were removed under reduced pressure. The residue was purified by preparative thin‐layer chromatography on silica gel by using hexane/ethyl acetate 2:1 mixture (*R*_f_=0.36) as eluent to obtain the Michael adduct as pale‐yellow crystals. Yields and enantiomeric excess (*ee*) values can be seen in Tables 3–4. These products had the same spectroscopic data as those reported (the absolute configuration was determined by the optical rotation of the products).4c

Finally, the catalyst in the centrifuge tubes was suspended in an appropriate solvent by using an ultrasonic bath and transferred into the reaction container to be reused in the next round.

HPLC: Phenomonex Lux Cellulose‐1 column (5 μm, 250×4.6 mm), eluent hexane/ethanol 85:15, isocratic mode; 0.8 mL min^−1^; temperature 20 °C, UV detector 254 nm. Retention time for (*S*)‐**15**: 16.1 min, for (*R*)‐**15**: 17.6 min. The amounts of the catalysts and reaction times are shown in Tables 3–4.

## Conflict of interest

The authors declare no conflict of interest.

## Supporting information

As a service to our authors and readers, this journal provides supporting information supplied by the authors. Such materials are peer reviewed and may be re‐organized for online delivery, but are not copy‐edited or typeset. Technical support issues arising from supporting information (other than missing files) should be addressed to the authors.

Supplementary
