# Supplementary material for: Synthesis and Applications of Cinchona Squaramide‐Modified Poly(Glycidyl Methacrylate) Microspheres as Recyclable Polymer‐Grafted Enantioselective Organocatalysts
Source: Chemistry. 2020 Sep 23;26(59):13513–22. doi: 10.1002/chem.202001993 (PMC7702047; doi:10.1002/chem.202001993)
Supplement: Supplementary file 1 — Supplementary [file CHEM-26-13513-s001.pdf]

Chemistry–A European Journal

Supporting Information

Synthesis and Applications of Cinchona Squaramide-Modified Poly(Glycidyl Methacrylate) Microspheres as Recyclable Polymer-Grafted Enantioselective Organocatalysts

Sándor Nagy,[a] Zsuzsanna Fehér,[a] Levente Kárpáti,[b, c] Péter Bagi,[a] Péter Kisszékelyi,[a] Béla Koczka,[d] Péter Huszthy,[a] Béla Pukánszky,[b] and József Kupai*[a]

Author Contributions

S.N. Conceptualization: Lead; Data curation: Lead; Investigation: Lead; Methodology: Lead; Visualization: Lead; Writing - Original Draft: Lead
Z.F. Data curation: Equal; Investigation: Lead; Methodology: Lead; Writing - Review & Editing: Equal
L.K. Conceptualization: Lead; Investigation: Equal; Methodology: Equal; Validation: Equal; Writing - Review & Editing: Equal
P.B. Data curation: Lead; Methodology: Equal; Writing - Review & Editing: Equal
P.K. Conceptualization: Lead; Validation: Equal; Writing - Review & Editing: Lead
B.K. Data curation: Equal
P.H. Funding acquisition: Lead; Writing - Review & Editing: Equal
B.P. Conceptualization: Equal; Funding acquisition: Equal; Writing - Review & Editing: Equal
J.K. Conceptualization: Lead; Funding acquisition: Equal; Project administration: Lead; Resources: Lead; Supervision: Lead; Writing - Original Draft: Equal; Writing - Review & Editing: Lead.

Supporting Information

1, SEM images and compositions of polymerization reactions 2

2, NMR spectra........ 9

NMR spectra of precatalyst **1** 9

NMR spectra of precatalyst **2** 11

NMR spectra of precatalyst **3** 16

NMR spectra of **10** 22

NMR spectra of **12** 26

3, Chiral HPLC profiles of *Michael* adducts **15** 29

1, SEM images and compositions of polymerization reactions

All SEM images were recorded on a JEOL JSM-5500LV scanning electron microscope in high vacuum at appropriate accelerating voltage (see in the images). Samples were coated with gold nanofilm layer by a vacuum nebulizer. The size distribution of microspheres was determined using Image-Pro Plus software. The elemental analysis of the samples was carried out without gold nanolayer using energy dispersive X-ray analysis (EDX with Si(Li) detector) applying 15 kV accelerating voltage and sampling time of 40 s.

Table S1. The applied compositions during the preparation of 'basic' microspheres, and the corresponding yields.

Compound	Composition (wt%)	
	I.[1]	II.
GMA	17.65	10.00
AIBN	0.35	0.10
PVP	2.65	4.00
MeOH	79.35	85.90
∑	100.00	100.00
**Yield at 50 °C (%)**	72[a]	68[a]
**Yield at 60 °C (%)**	70[a]	89
**Yield at 65 °C (%)**	90	92

[a] The polymerizations applying composition I. or II. at 50 °C, and applying composition I. at 60 °C gave aggregates. As an example, see Figure S1.

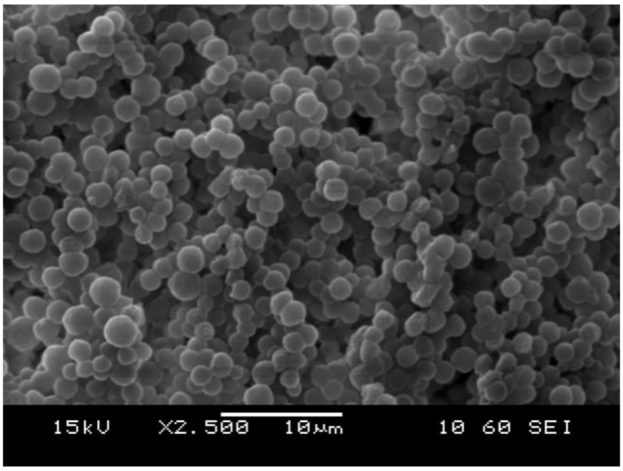

Figure S1. SEM image of microsphere aggregates prepared at 50 °C applying composition II.

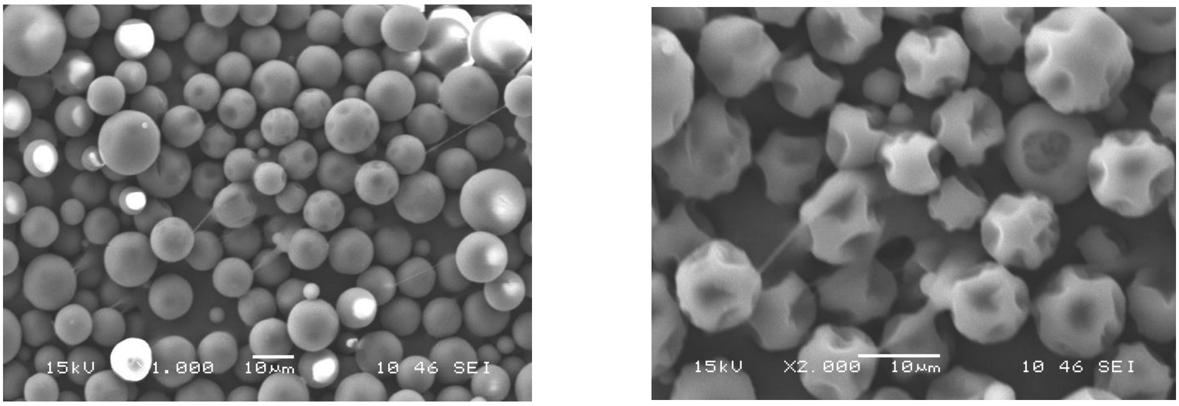

Figure S2. SEM image of microspheres with wide size distribution prepared at 65 °C applying *Composition II.* Using PVP10 (1000× magnification left, 2000× magnification right).

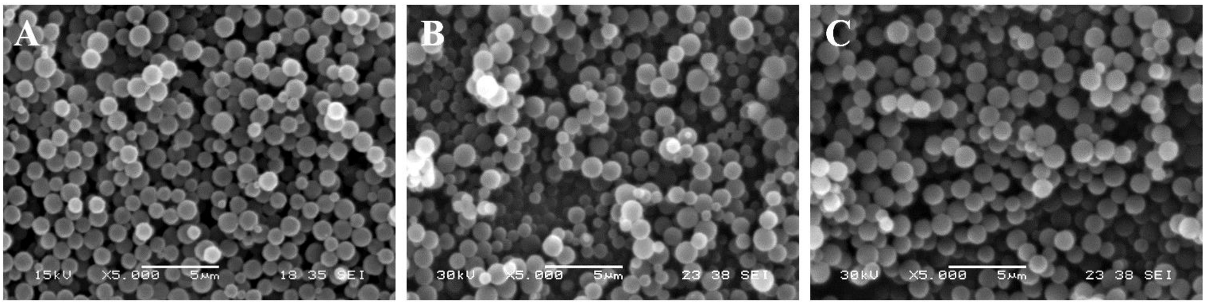

Figure S3. SEM image of microspheres after the modification with **C2** (A), **C2**-modified PGMA after five runs in EtOAc (B) and in DCM (C).

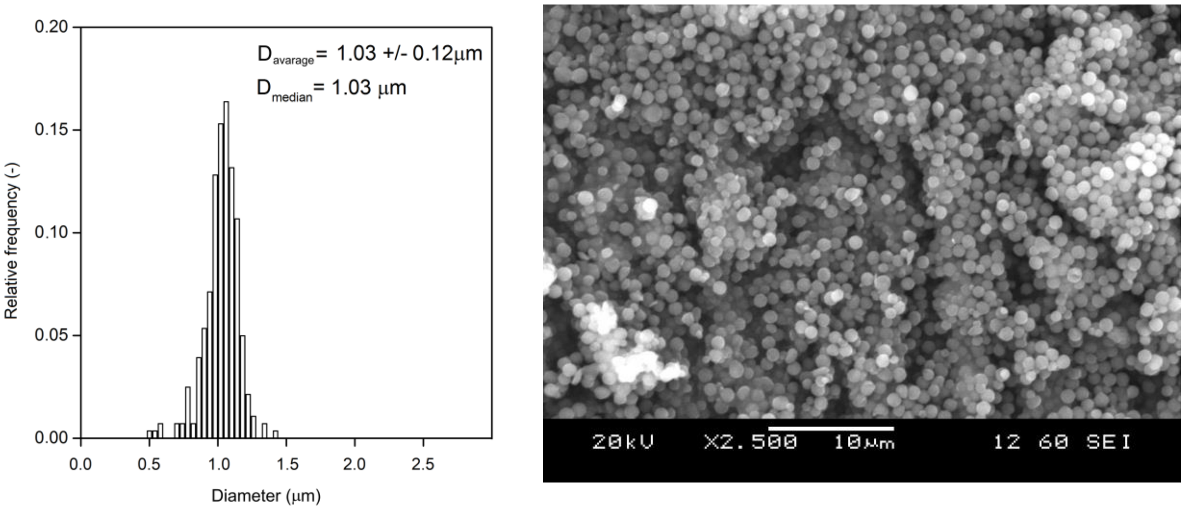

Figure S4. Size distribution and SEM image of microspheres prepared at 60 °C applying composition II.

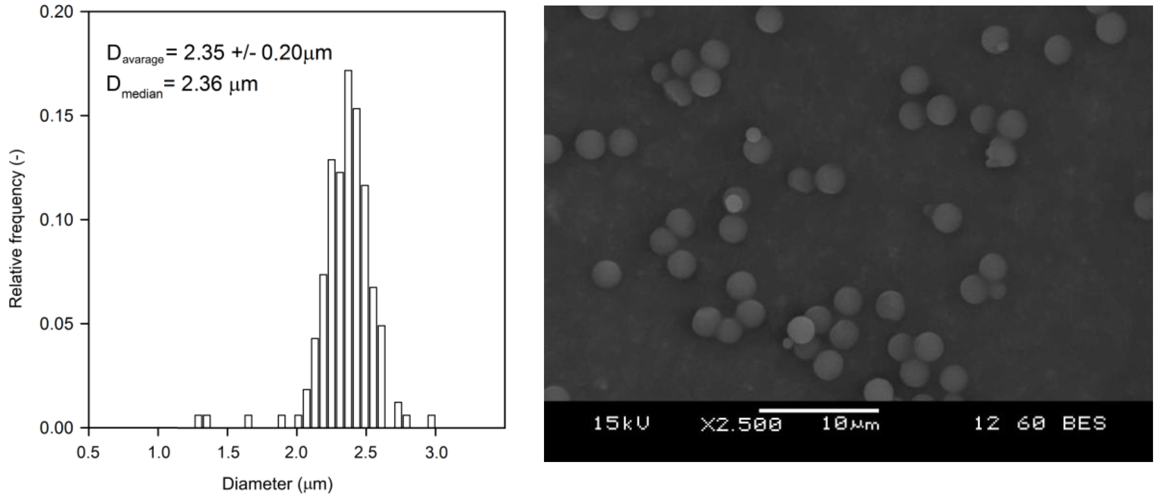

Figure S5. Size distribution and SEM image of microspheres prepared at 65 °C applying composition I.

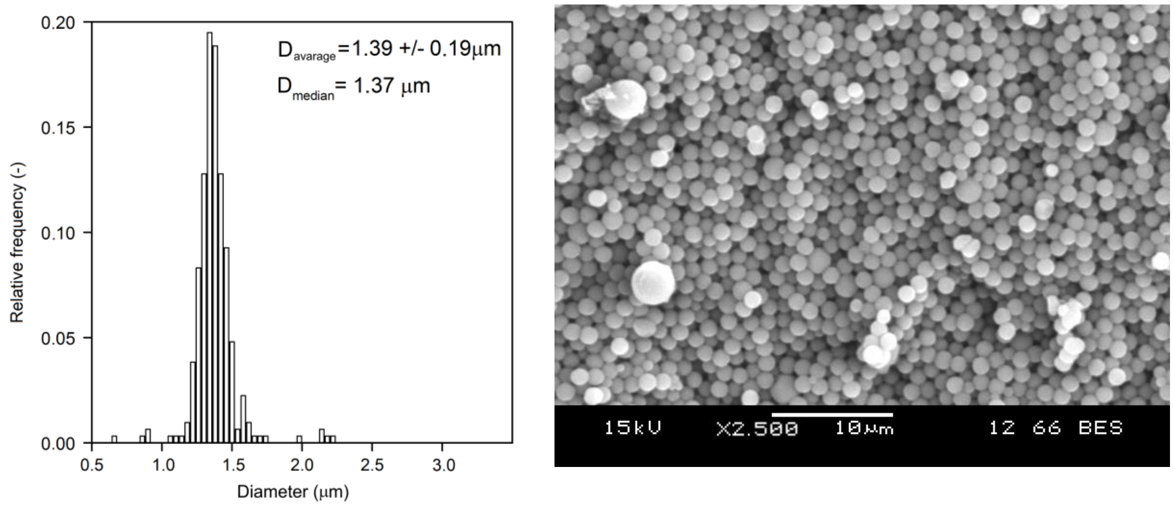

Figure S6. Size distribution and SEM image of microspheres prepared at 65 °C applying composition II.

Table S2. Effect of PVP wt% on the polymerization at 65 °C with different compositions.

Compound	Composition (wt%)				
	III.	IV.	V.	VI.	II.
GMA	10.0	10.0	10.0	10.0	10.0
AIBN	0.1	0.1	0.1	0.1	0.1
**PVP**	**1.0**	**1.4**	**2.0**	**2.5**	**4.0**
MeOH	88.9	88.5	87.9	87.4	85.9
∑	100.0	100.0	100.0	100.0	100.0
**Yield (%)**	85	92	93	88	92

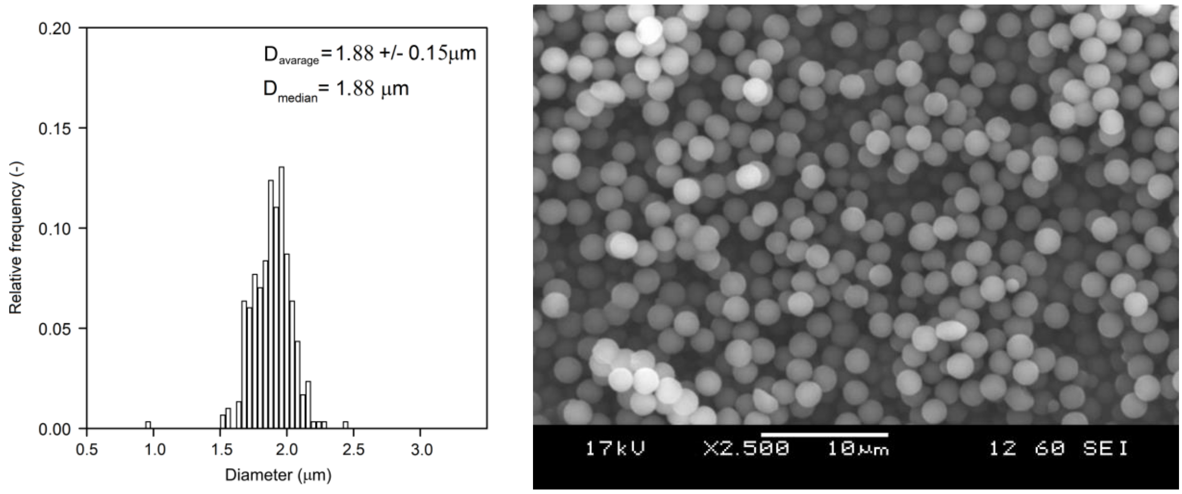

Figure S7. Size distribution and SEM image of microspheres prepared at 65 °C applying composition III.

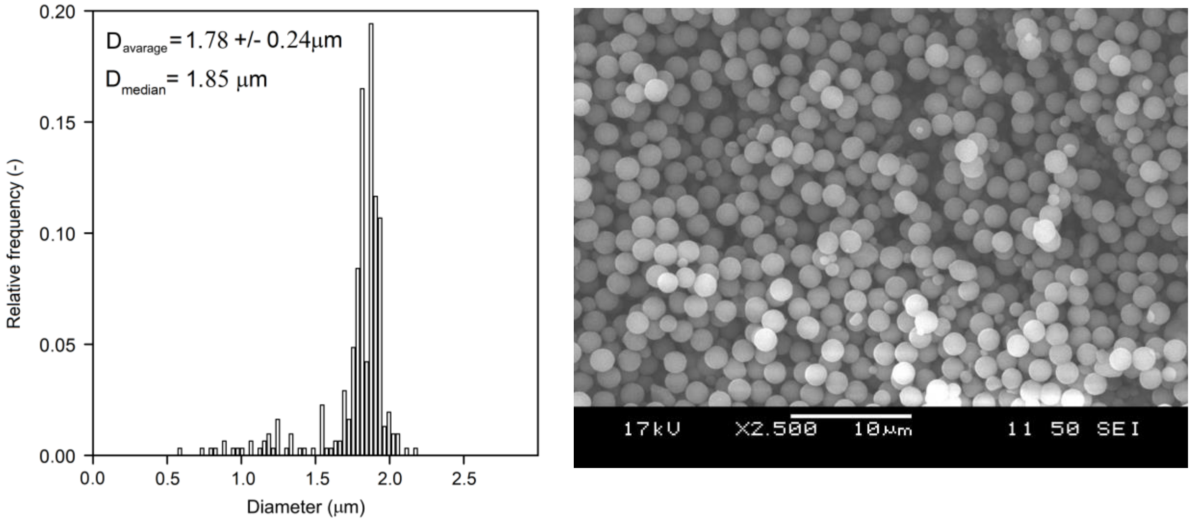

Figure S8. Size distribution and SEM image of microspheres prepared at 65 °C applying composition IV.

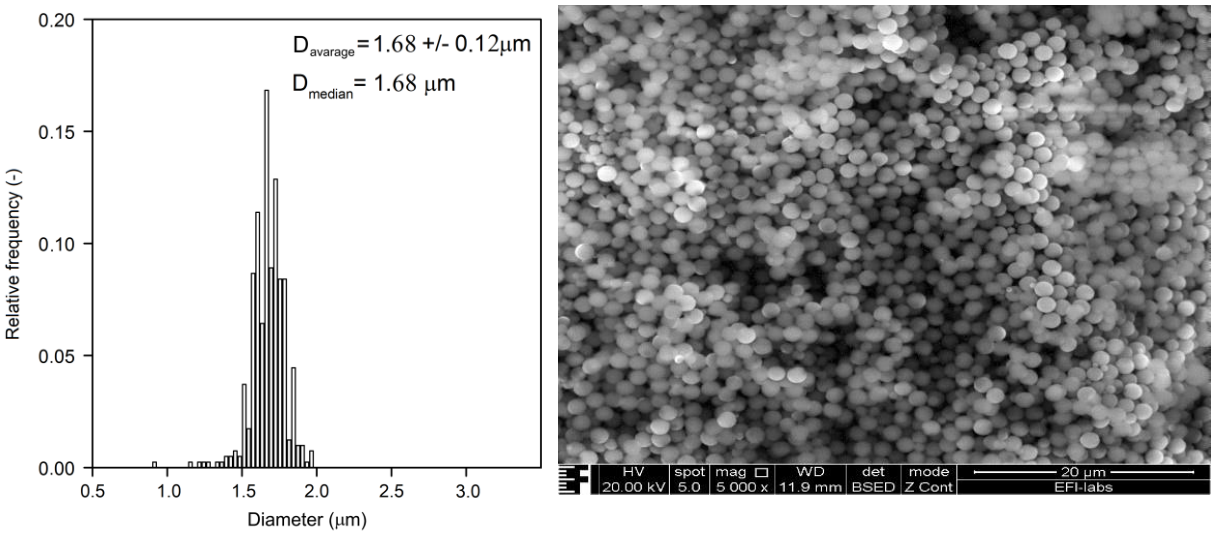

Figure S9. Size distribution and SEM image of microspheres prepared at 65 °C applying composition V.

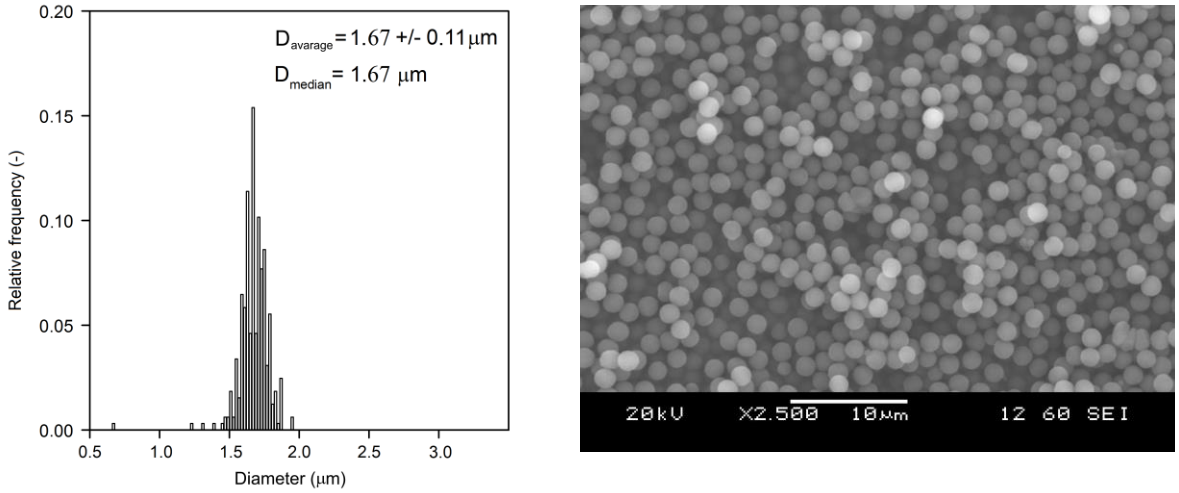

Figure S10. Size distribution and SEM image of microspheres prepared at 65 °C applying composition VI.

Table S3. Effect of EGDMA content on the subsequent polymerization at 60 °C with different compositions.

Compound	Composition (wt%)				
	VII.	VIII.	IX.	X.	XI.
PGMA	4.0	4.0	4.0	4.0	4.0
AIBN	0.1	0.1	0.1	0.1	0.1
**EGDMA**	**0.6**	**0.8**	**1.0**	**1.2**	**1.4**
PVP	4.0	4.0	4.0	4.0	4.0
MeOH	91.3	91.1	90.9	90.7	90.5
∑	100.0	100.0	100.0	100.0	100.0
**Yield (%)**	99	91	93	89	98

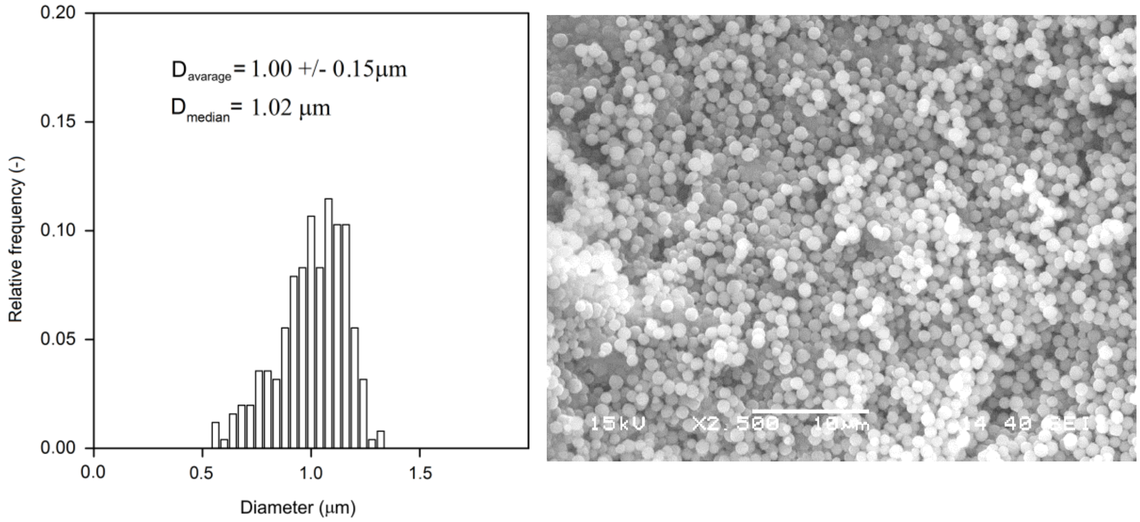

Figure S11. Size distribution and SEM image of crosslinked microspheres applying composition VII.

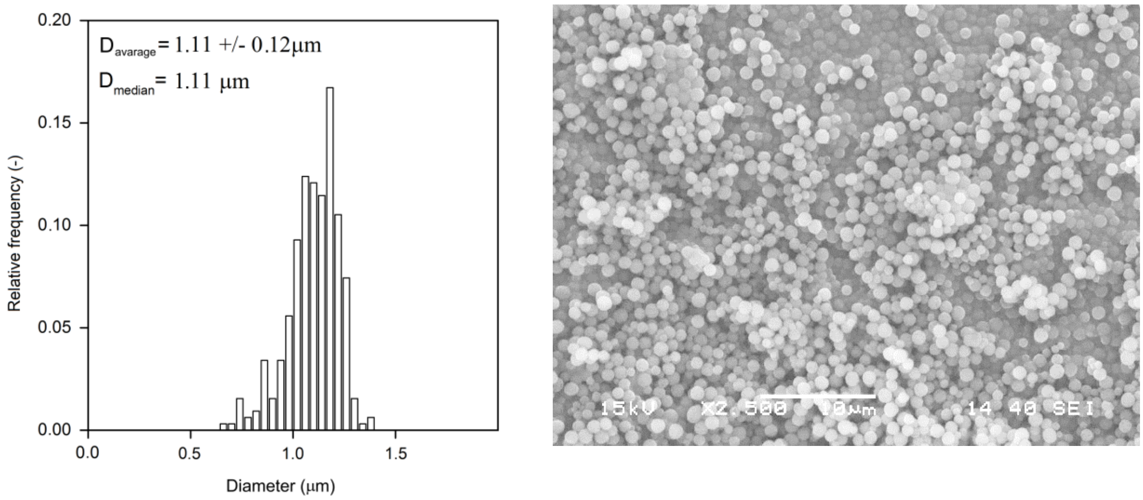

Figure S12. Size distribution and SEM image of crosslinked microspheres applying composition VIII.

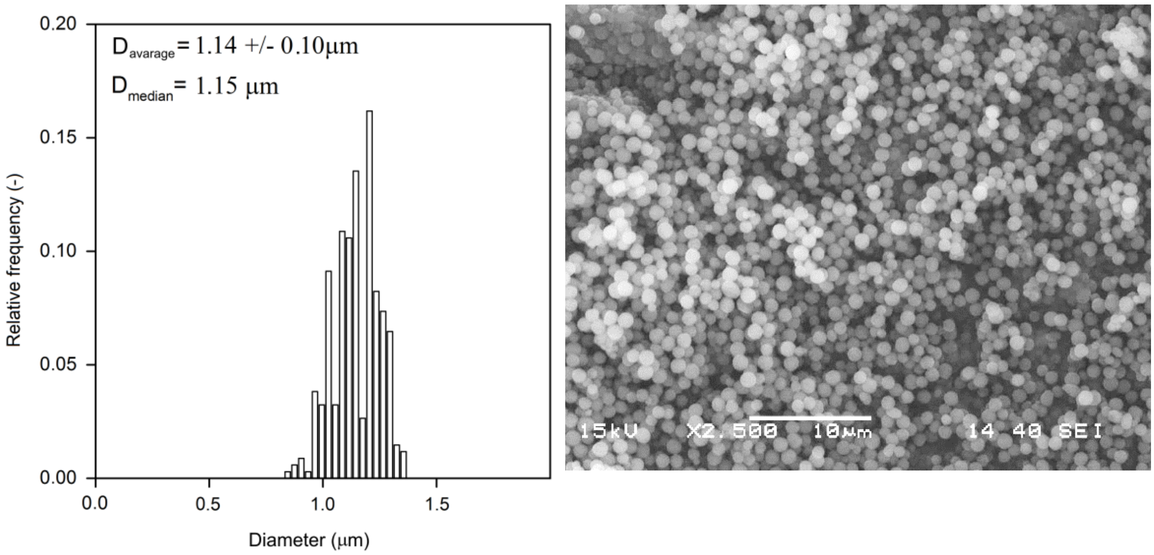

Figure S13. Size distribution and SEM image of crosslinked microspheres applying composition IX.

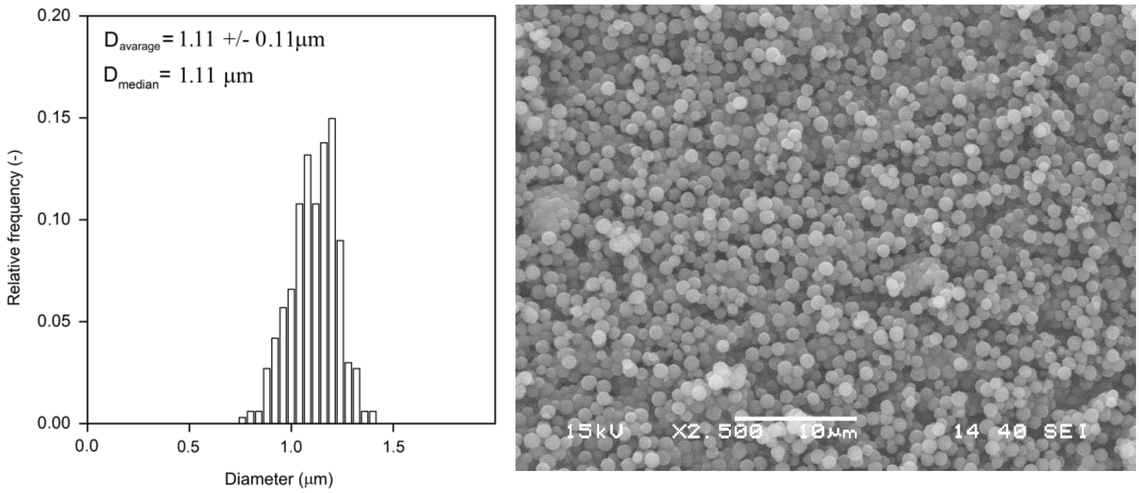

Figure S14. Size distribution and SEM image of crosslinked microspheres applying composition X.

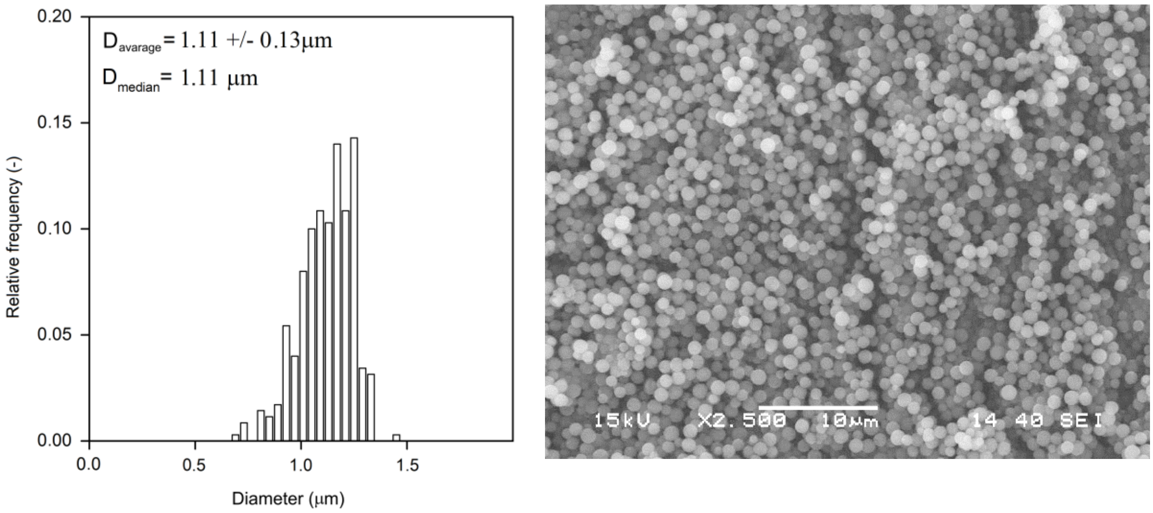

Figure S15. Size distribution and SEM image of crosslinked microspheres applying composition XI.

2, NMR spectra

NMR spectra of precatalyst **1**

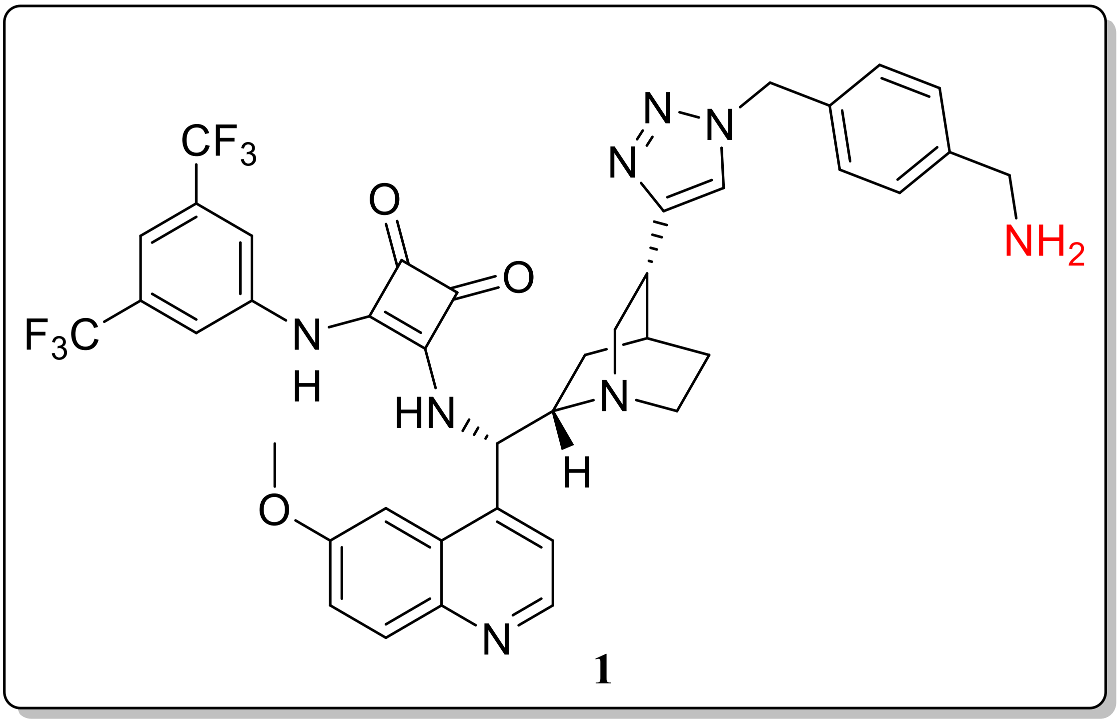

1

Figure S16. Molecule structure of precatalyst **1**

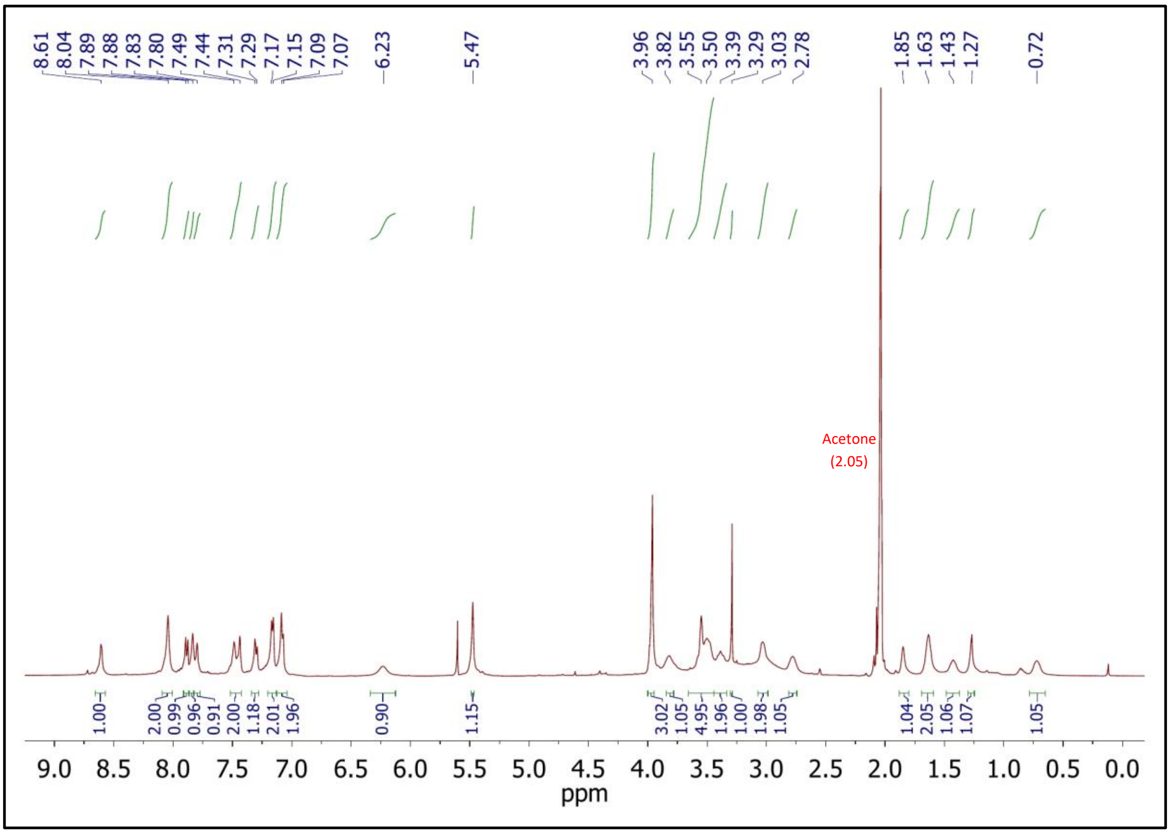

Figure S17a. Full ^{1}H NMR spectrum of precatalyst **1** (500 MHz, acetone-d_6)

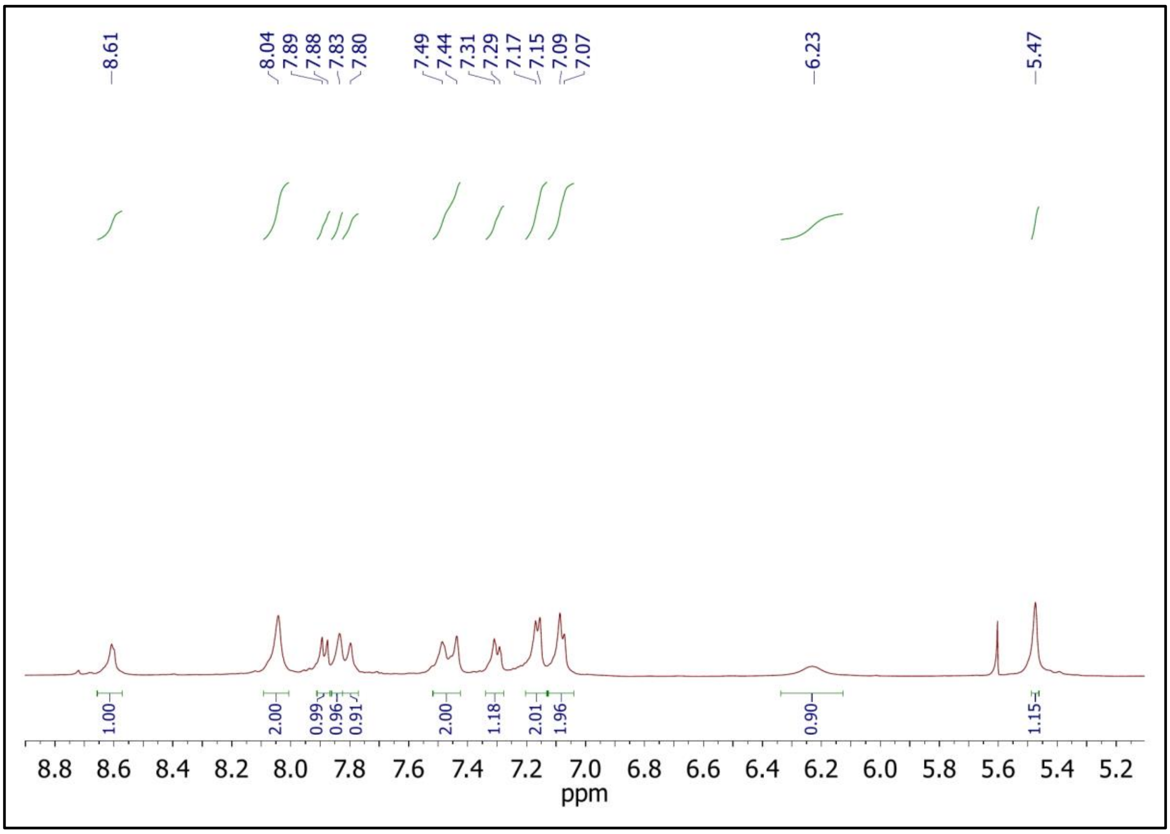

Figure S17b. Central region of ^{1}H NMR spectrum of precatalyst **1** (500 MHz, acetone-d_6)

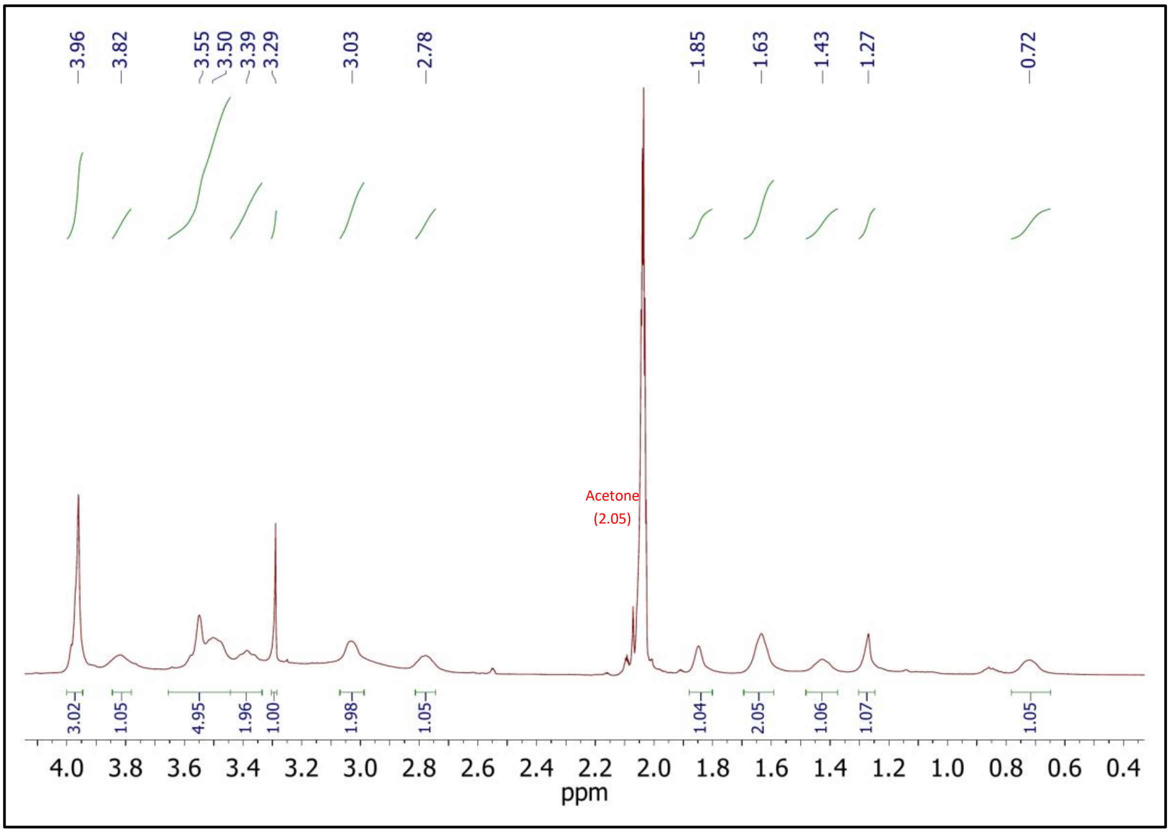

Figure S17c. Lower region of ^{1}H NMR spectrum of precatalyst **1** (500 MHz, acetone-d_6)

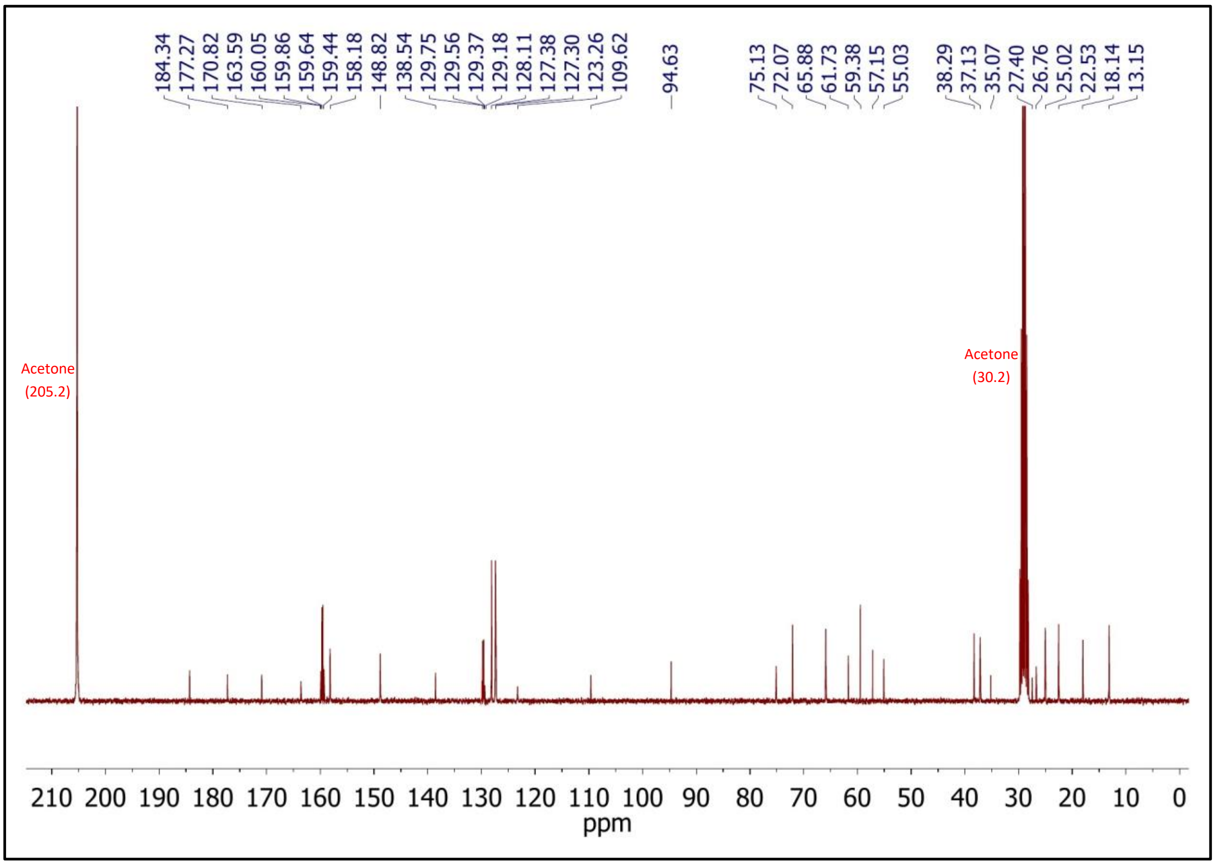

Figure S18. ^{13}C NMR spectrum of precatalyst **1** (125 MHz, acetone-d_6)

NMR spectra of precatalyst **2**

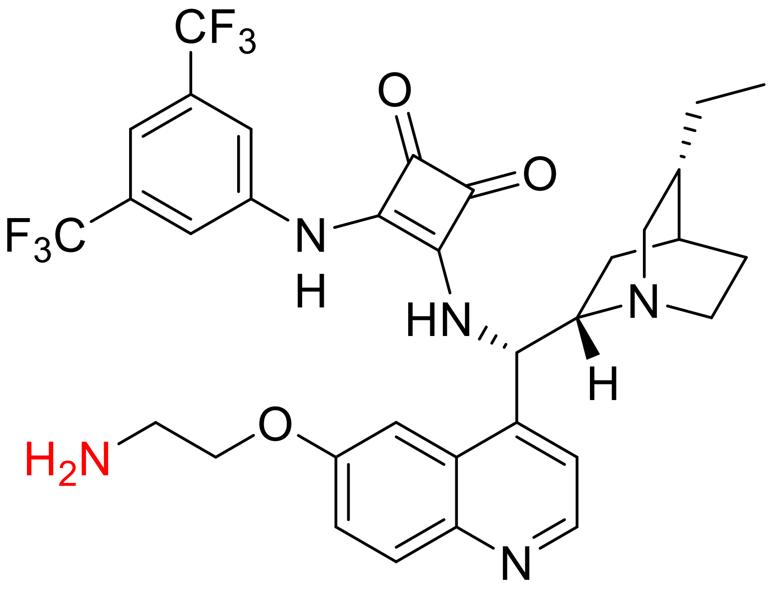

2

Figure S19. Molecule structure of precatalyst **2**

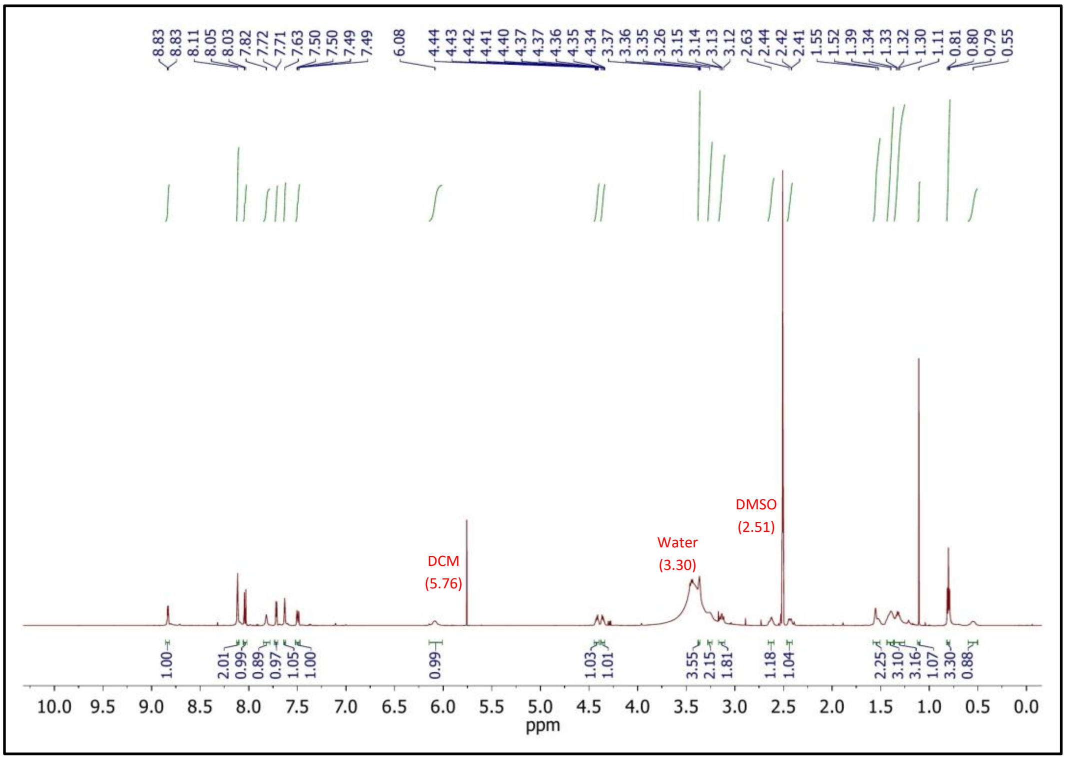

Figure S20a. Full ^{1}H NMR spectrum of precatalyst **2** (600 MHz, DMSO-d_6)

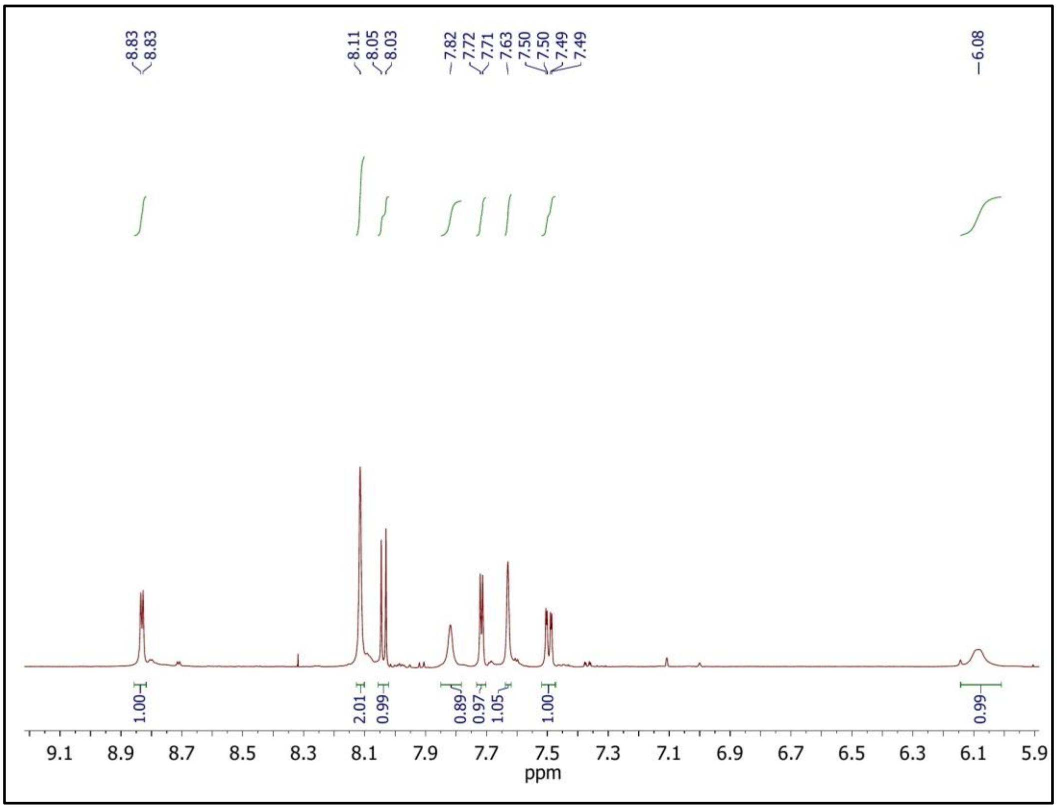

Figure S20b. Upper region of ^{1}H NMR spectrum of precatalyst **2** (600 MHz, DMSO-d_6)

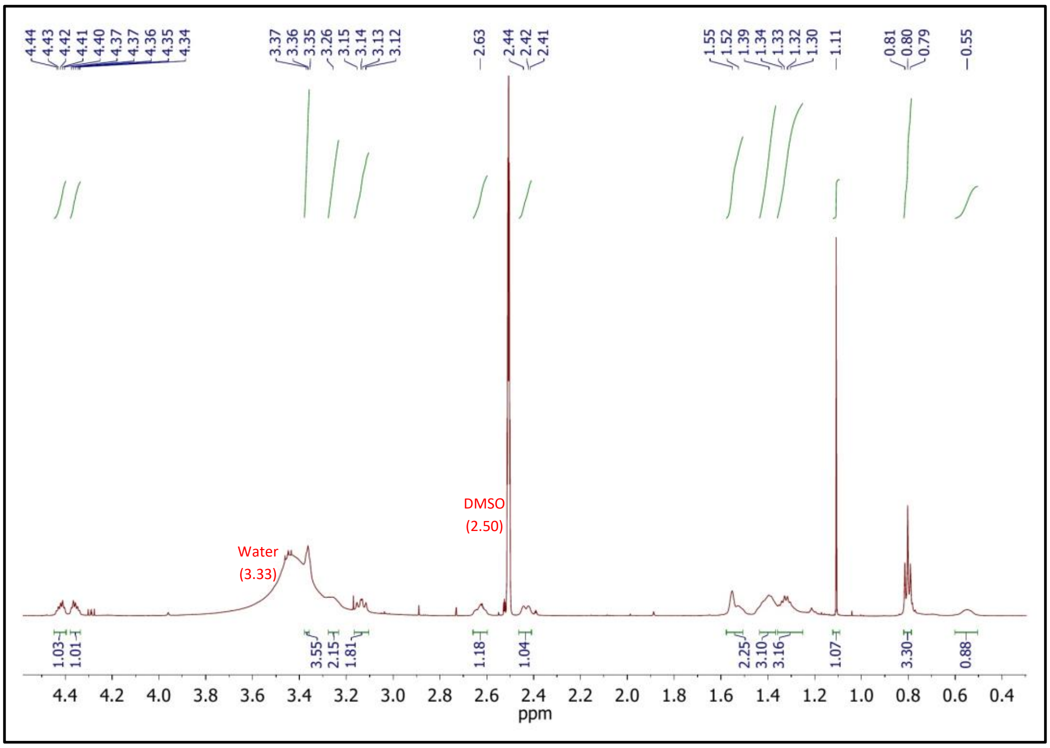

Figure S20c. Lower region of ^{1}H NMR spectrum of precatalyst **2** (600 MHz, DMSO-d_6)

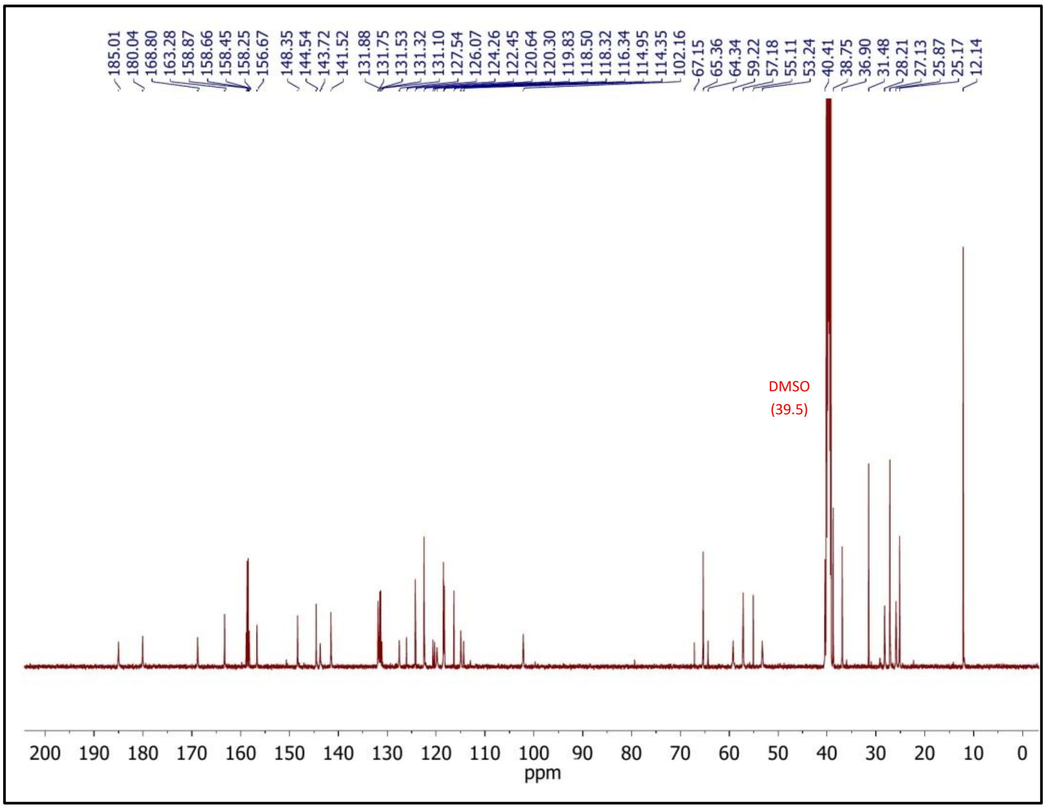

Figure S21. ^{13}C NMR spectrum of precatalyst **2** (150 MHz, DMSO-d_6)

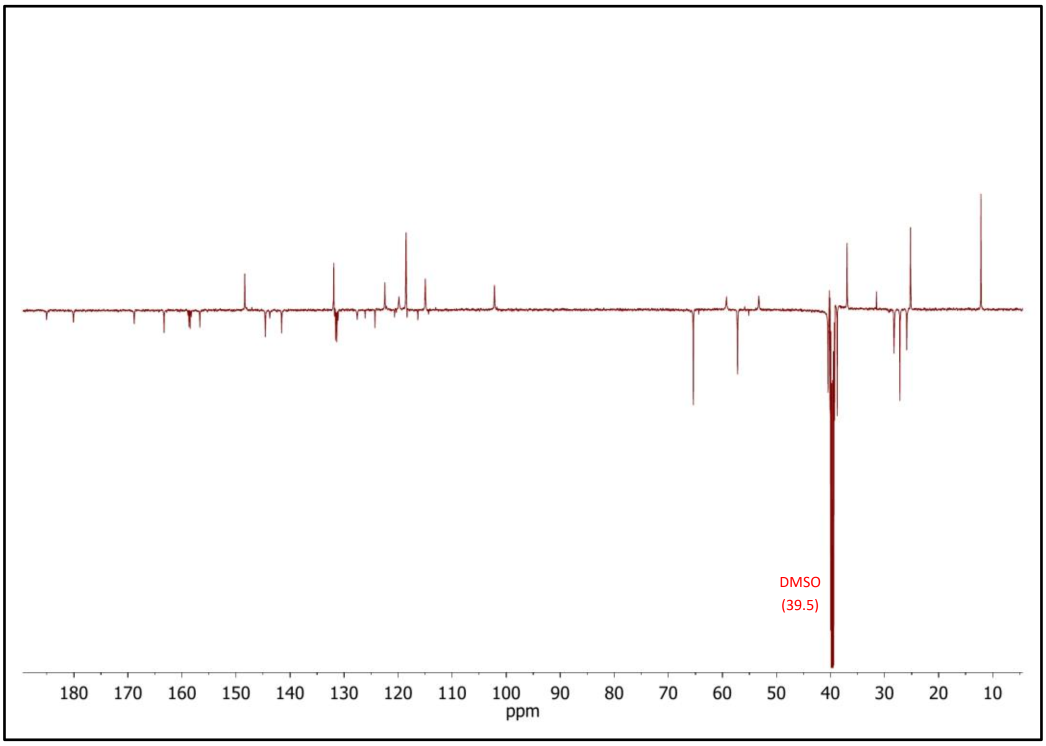

Figure S22. ^{13}C DEPT NMR spectrum of precatalyst **2** (DMSO-d_6)

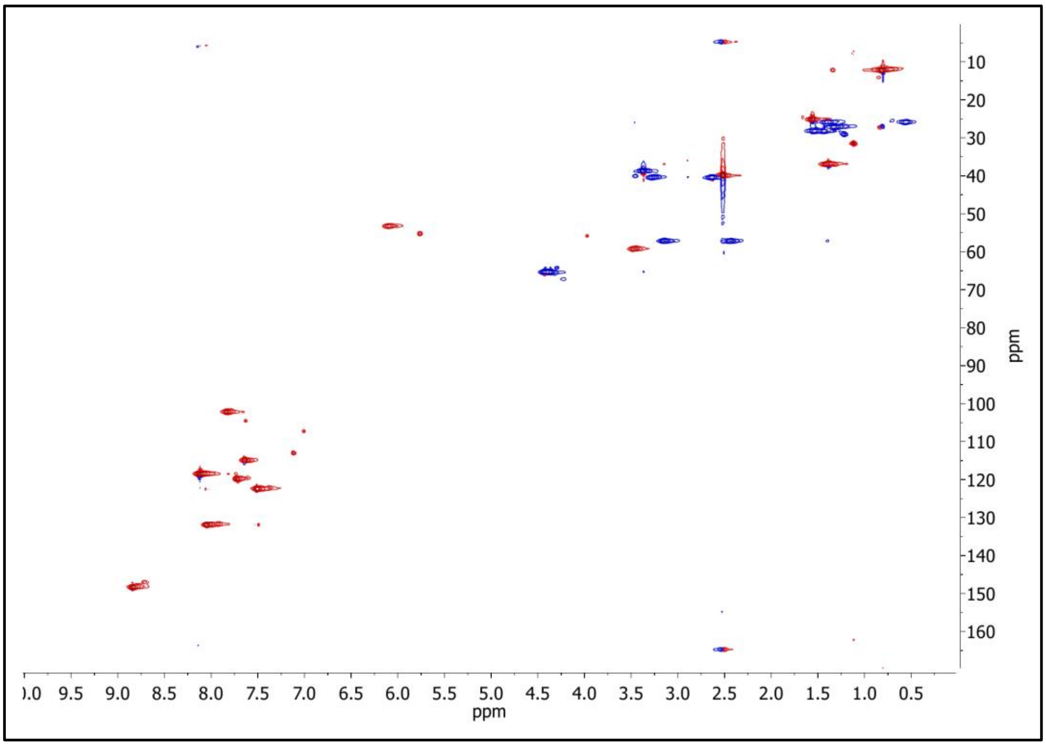

Figure S23. HSQC spectrum of precatalyst **2** (DMSO-d_6)

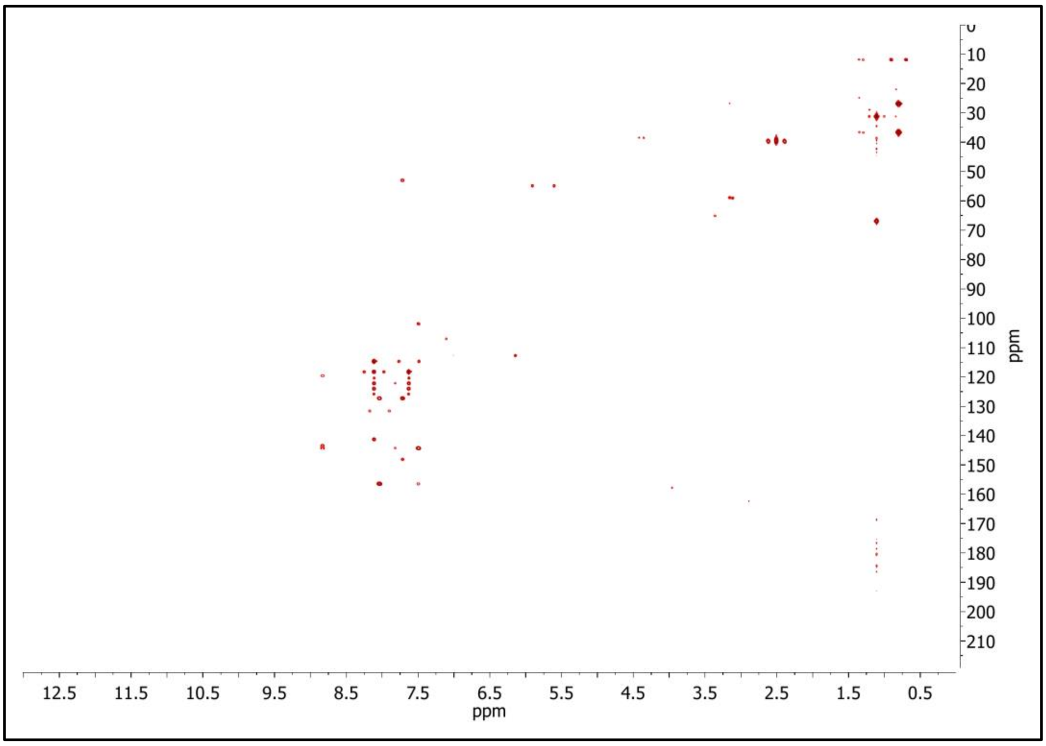

Figure S24. HMBC NMR spectrum of precatalyst **2** (DMSO-d$_6$)

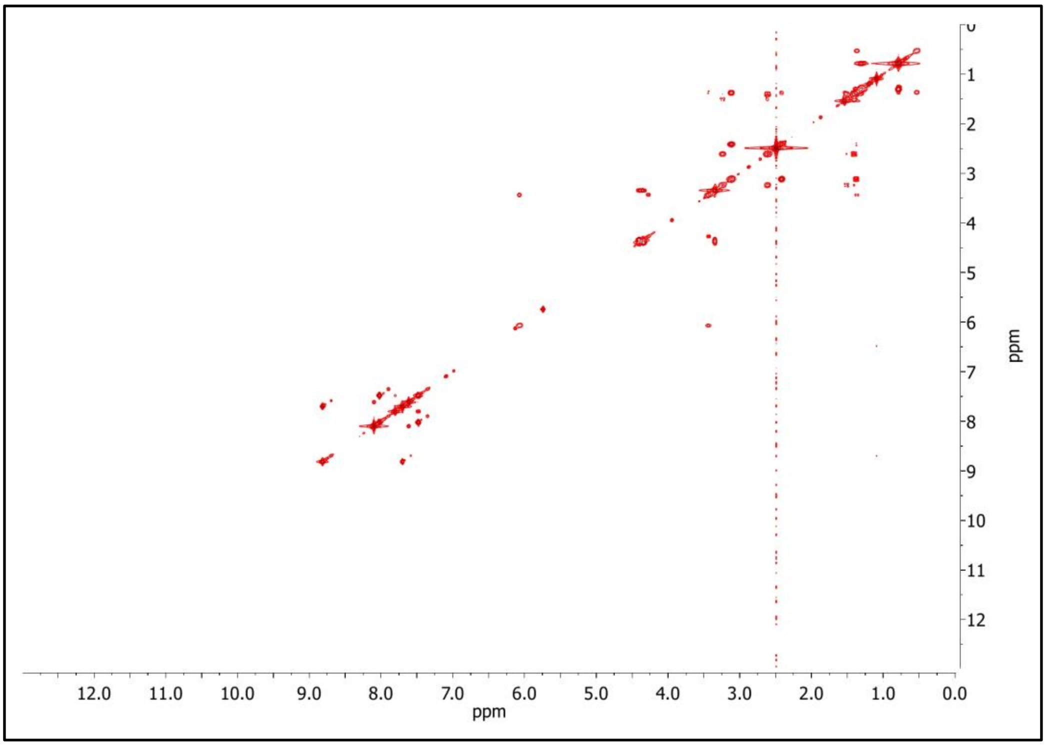

Figure S25. COSY NMR spectrum of precatalyst **2** (DMSO-d$_6$)

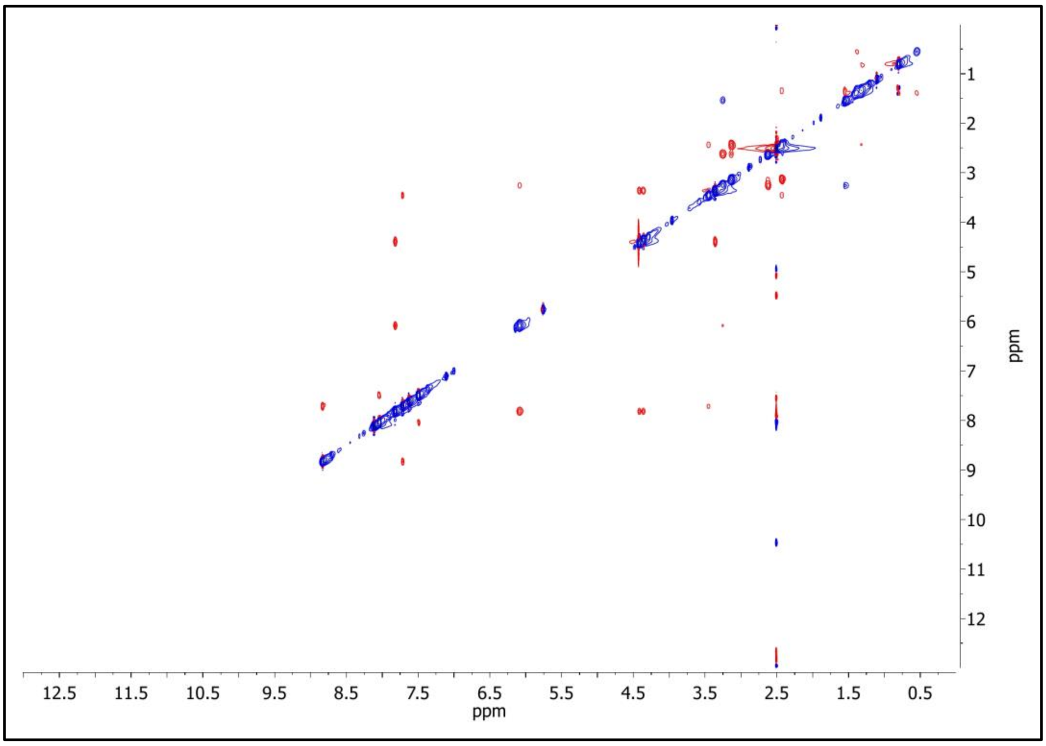

Figure S26. ROESY NMR spectrum of precatalyst **2** (DMSO-d_6)

NMR spectra of precatalyst **3**

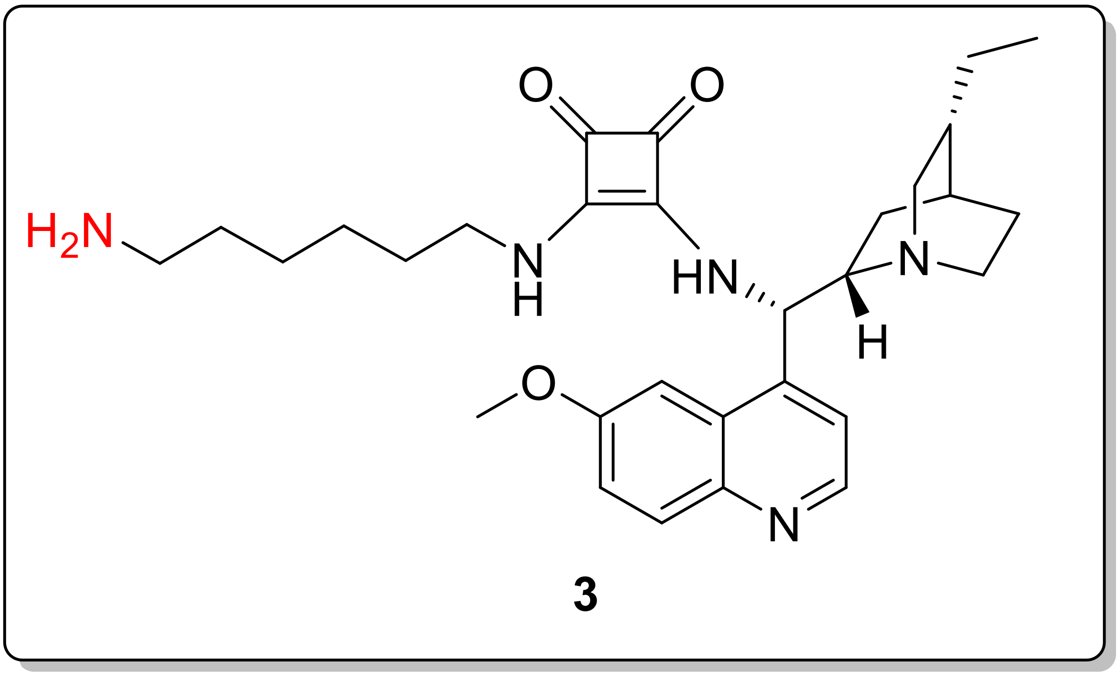

Figure S27. Molecule structure of precatalyst **3**

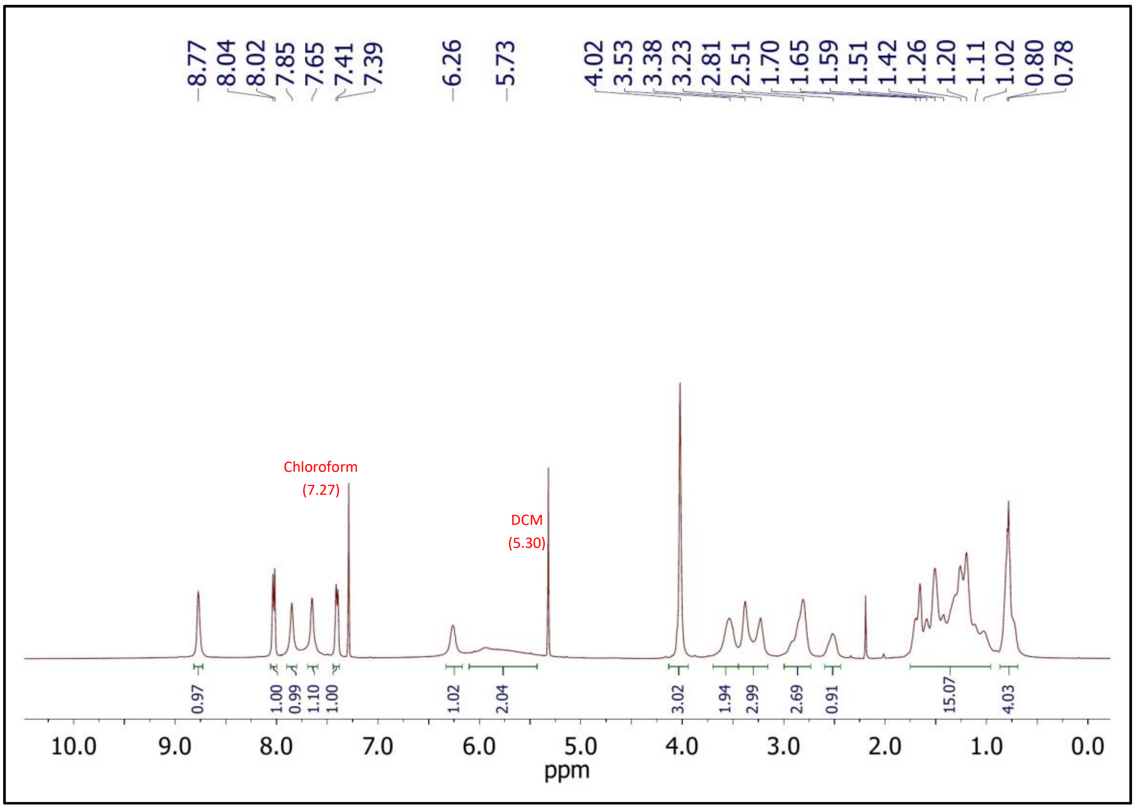

Figure S28a. Full ^{1}H NMR spectrum of precatalyst **3** (500 MHz, $CDCl_3$)

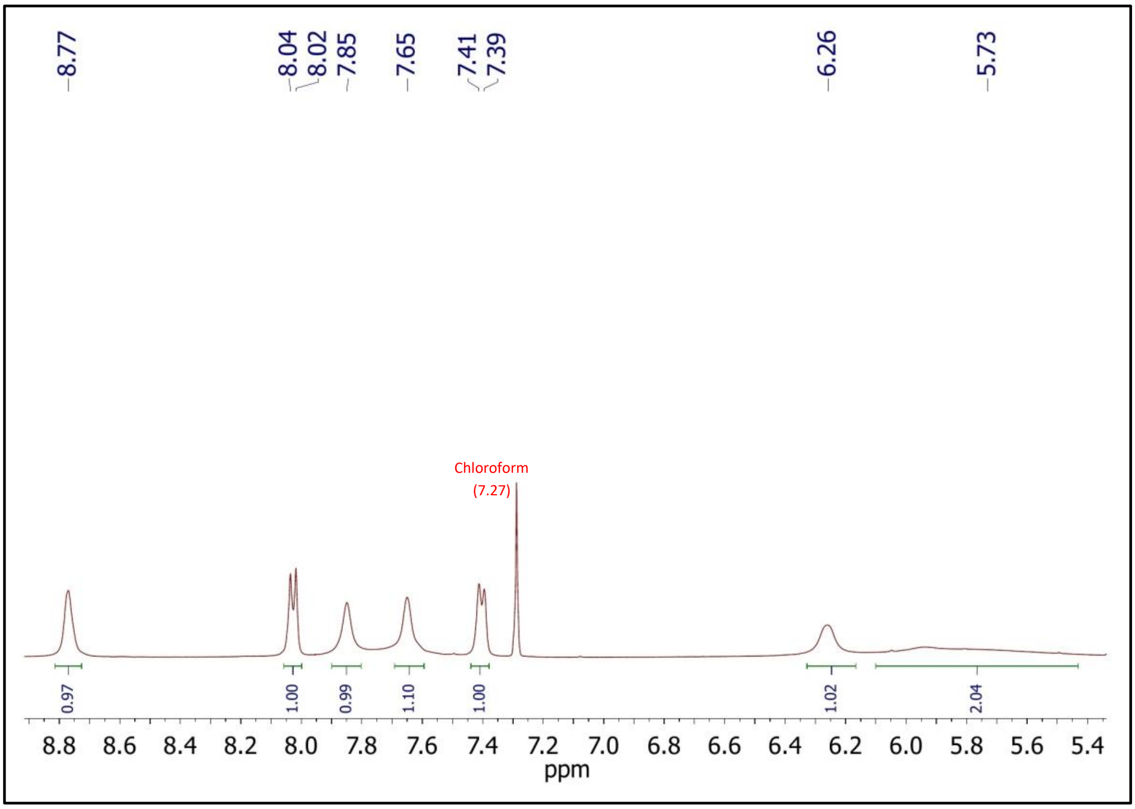

Figure S28b. Upper region of ^{1}H NMR spectrum of precatalyst **3** (500 MHz, $CDCl_3$)

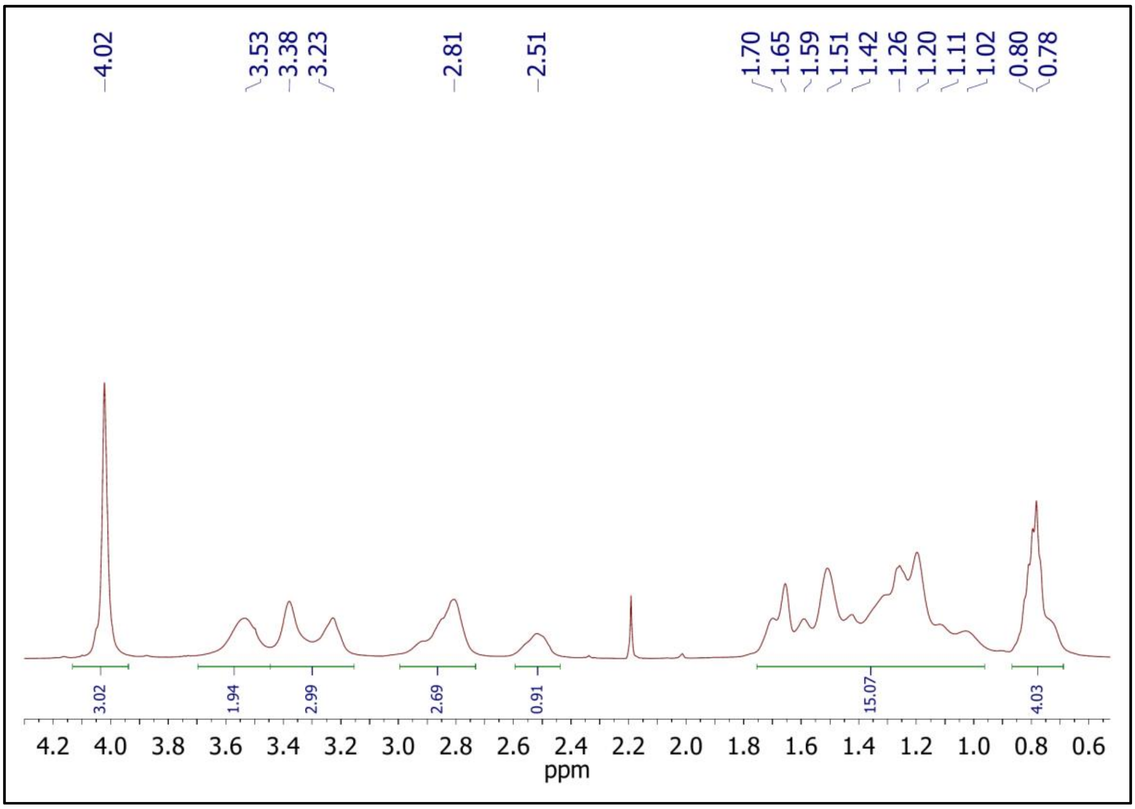

Figure S28c. Lower region of ^{1}H NMR spectrum of precatalyst **3** (500 MHz, $CDCl_3$)

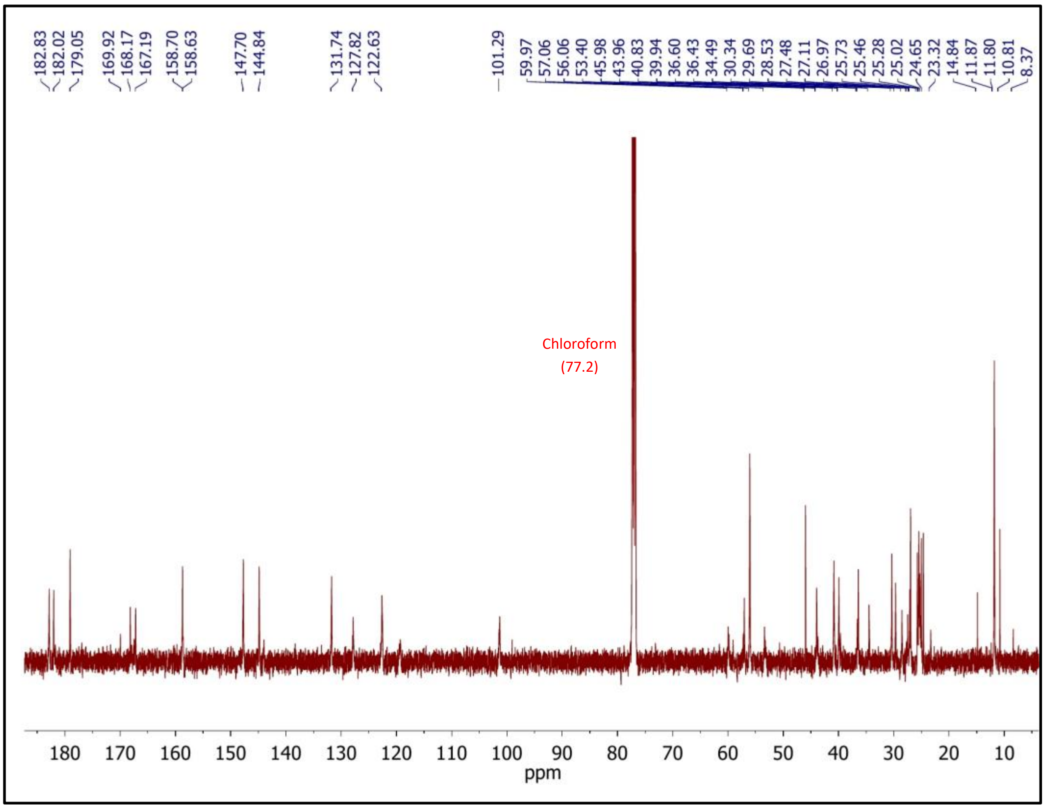

Figure S29. ^{13}C NMR spectrum of precatalyst **3** (125 MHz, $CDCl_3$)

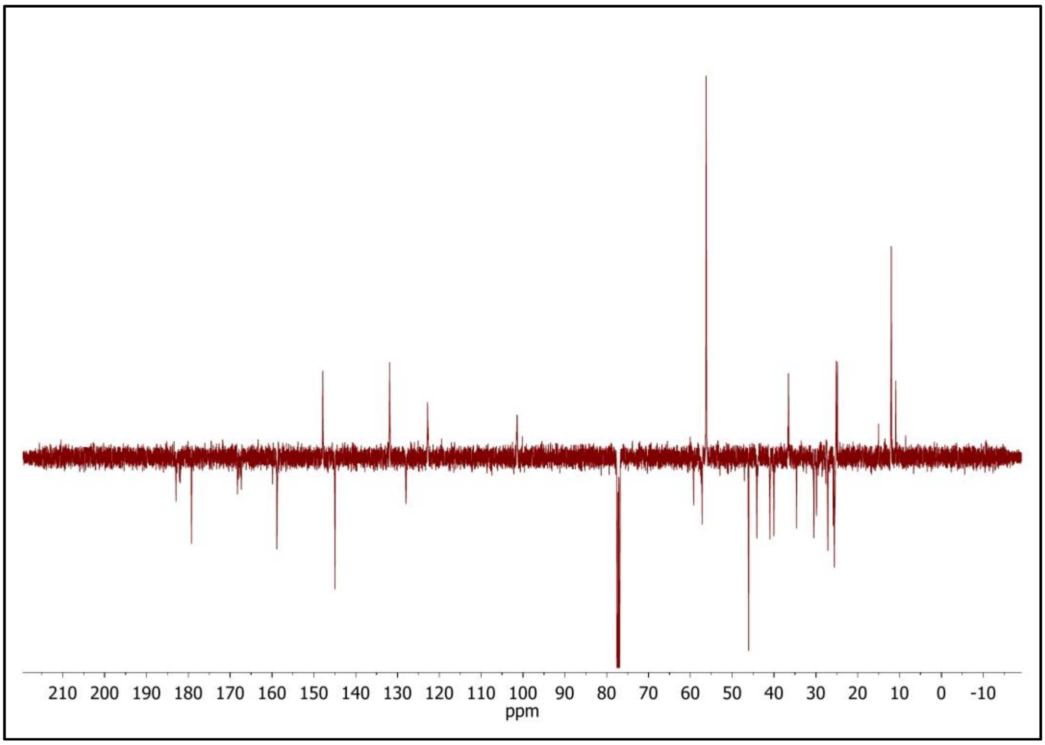

Figure S30. ^{13}C DEPT NMR spectrum of precatalyst **3** ($CDCl_3$)

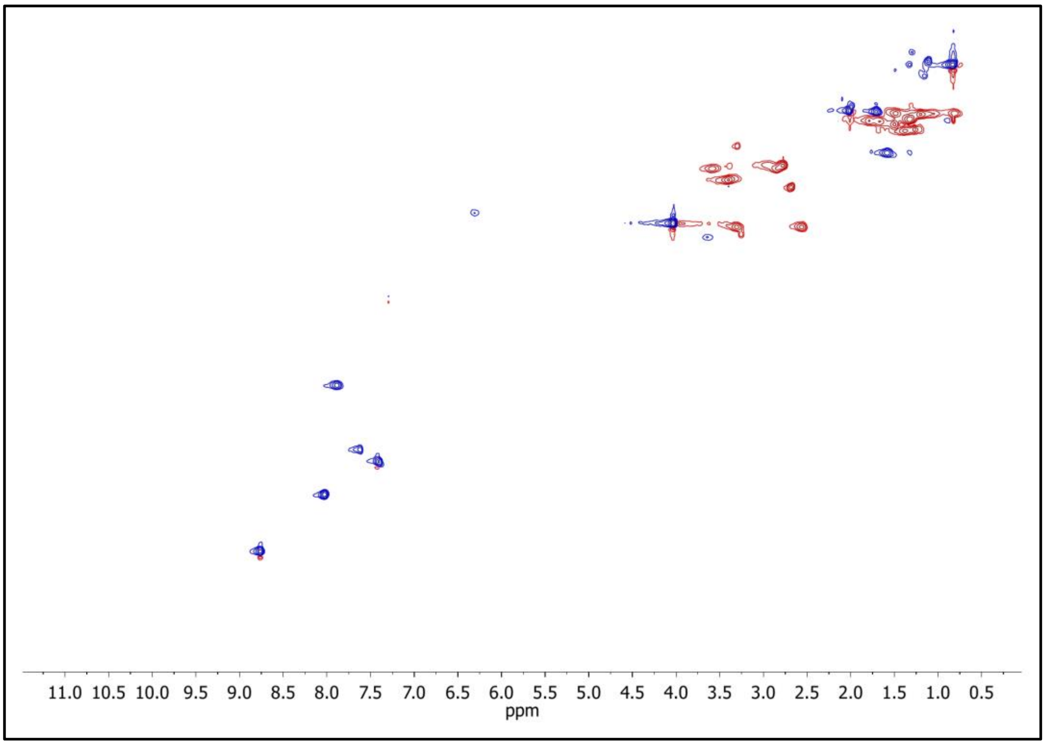

Figure S31. HSQC NMR spectrum of precatalyst **3** ($CDCl_3$)

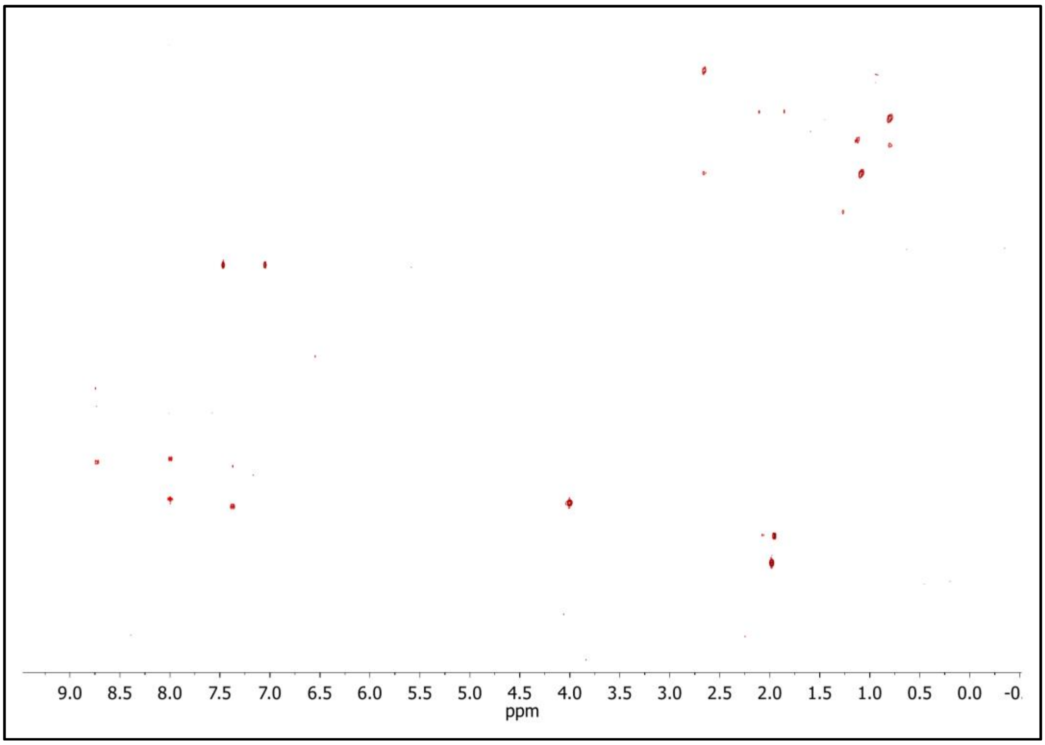

Figure S32. HMBC NMR spectrum of precatalyst **3** ($CDCl_3$)

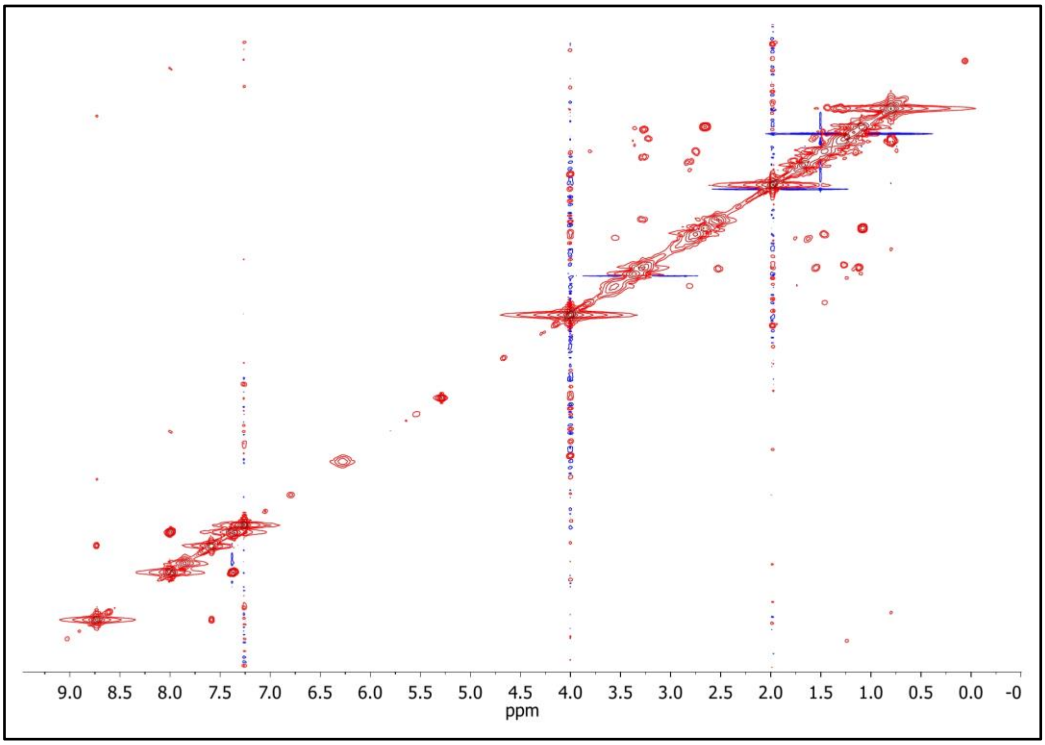

Figure S33. COSY NMR spectrum of precatalyst **3** ($CDCl_3$)

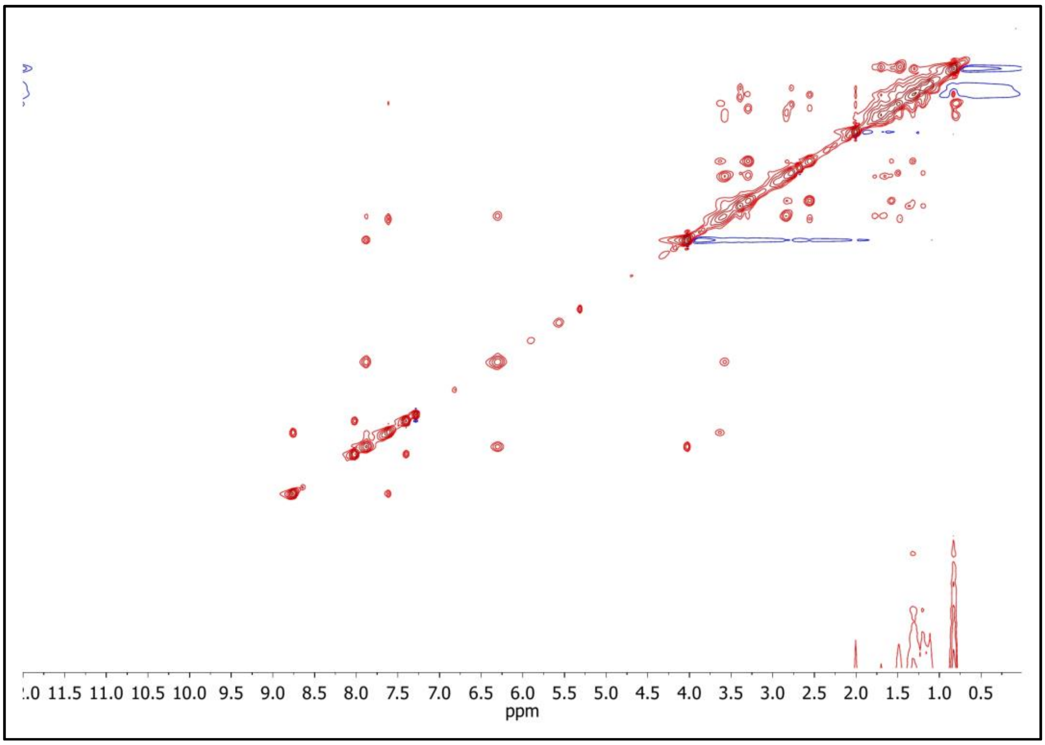

Figure S34. NOESY NMR spectrum of precatalyst **3** ($CDCl_3$)

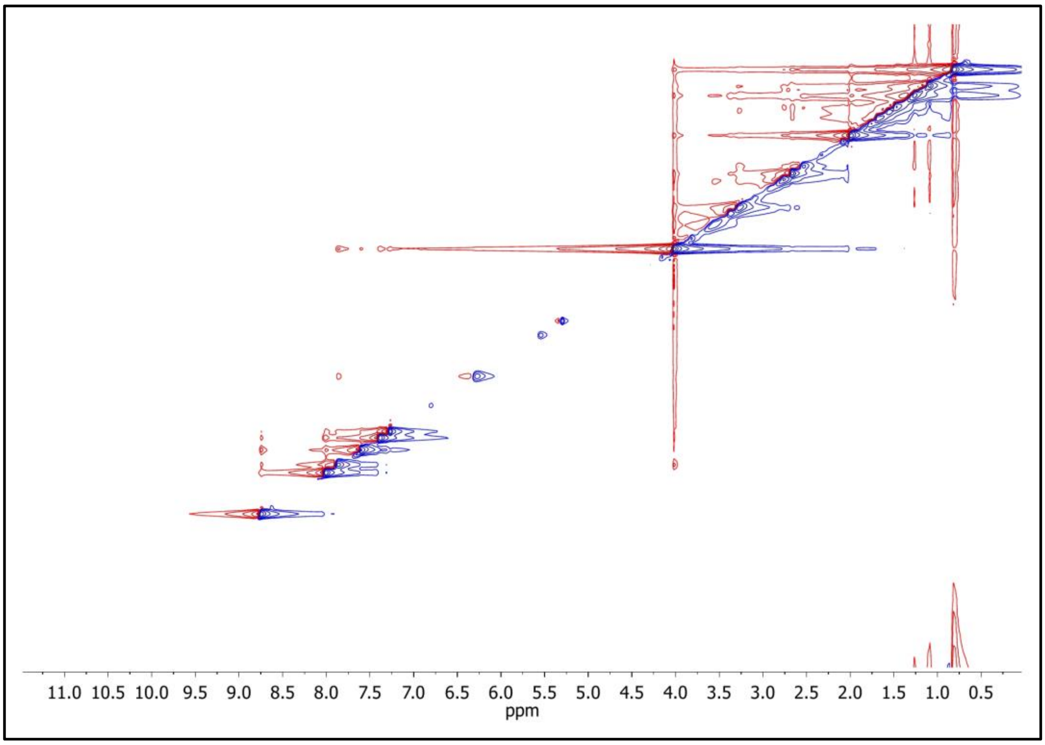

Figure S35. ROESY NMR spectrum of precatalyst **3** ($CDCl_3$)

NMR spectra of **10**

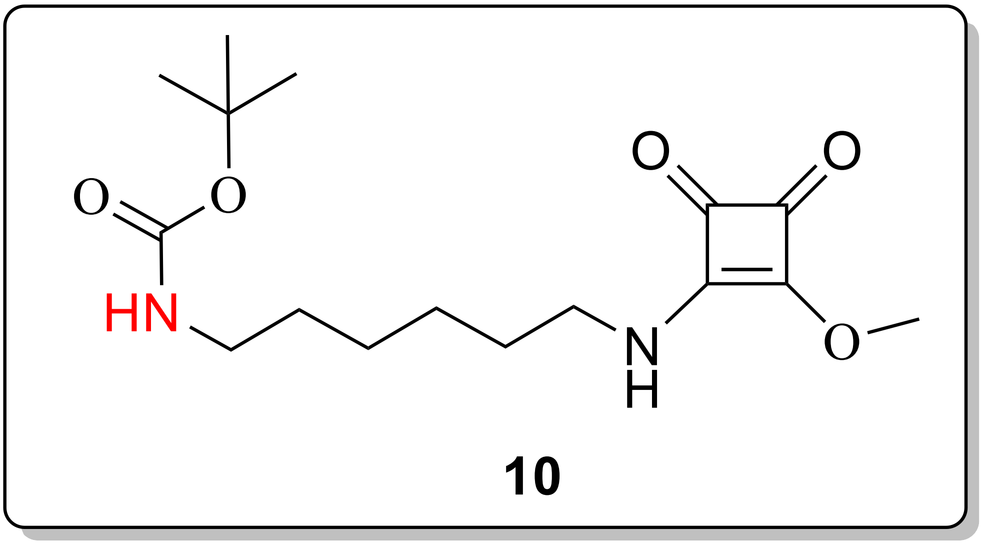

Figure S36. Molecule structure of **10**

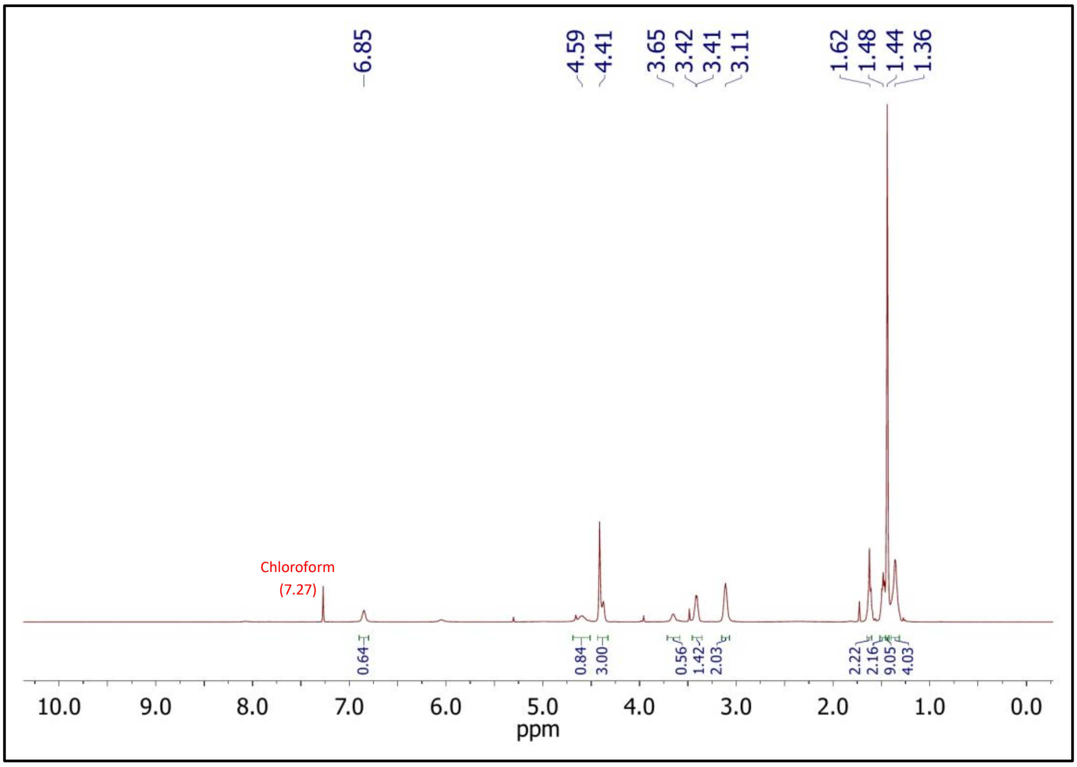

Figure S37a. Full ^{1}H NMR spectrum of **10** (500 MHz, $CDCl_3$)

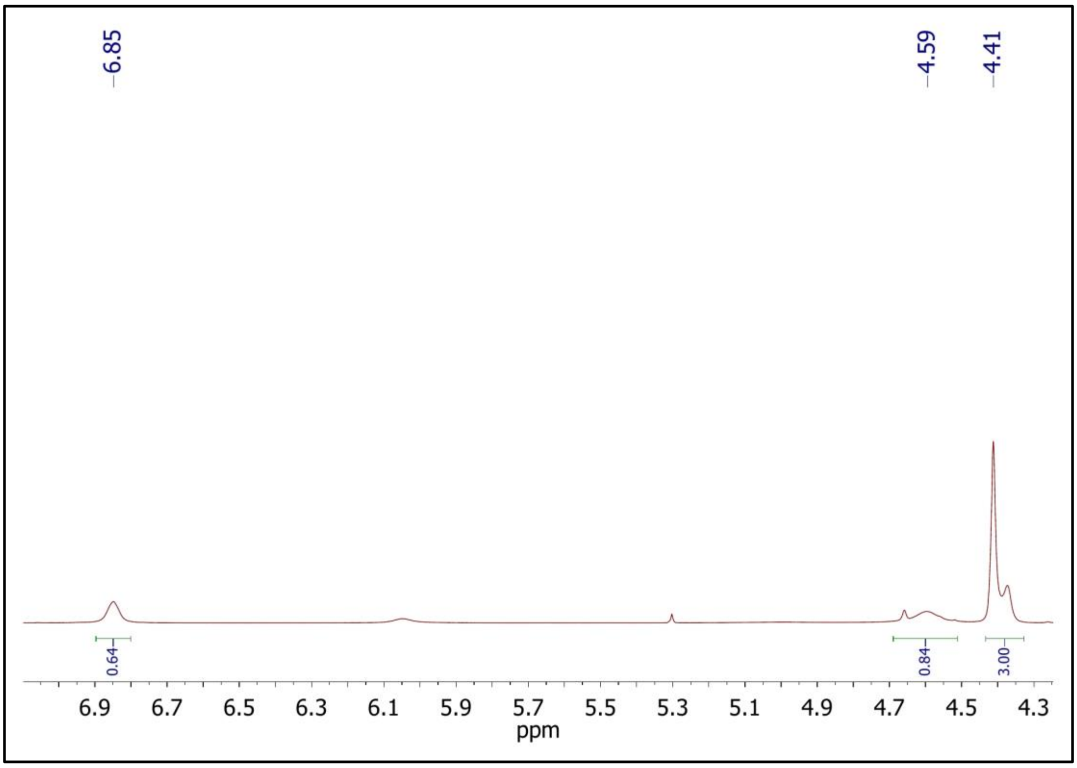

Figure S37b. Upper region of ^{1}H NMR spectrum of **10** (500 MHz, $CDCl_3$)

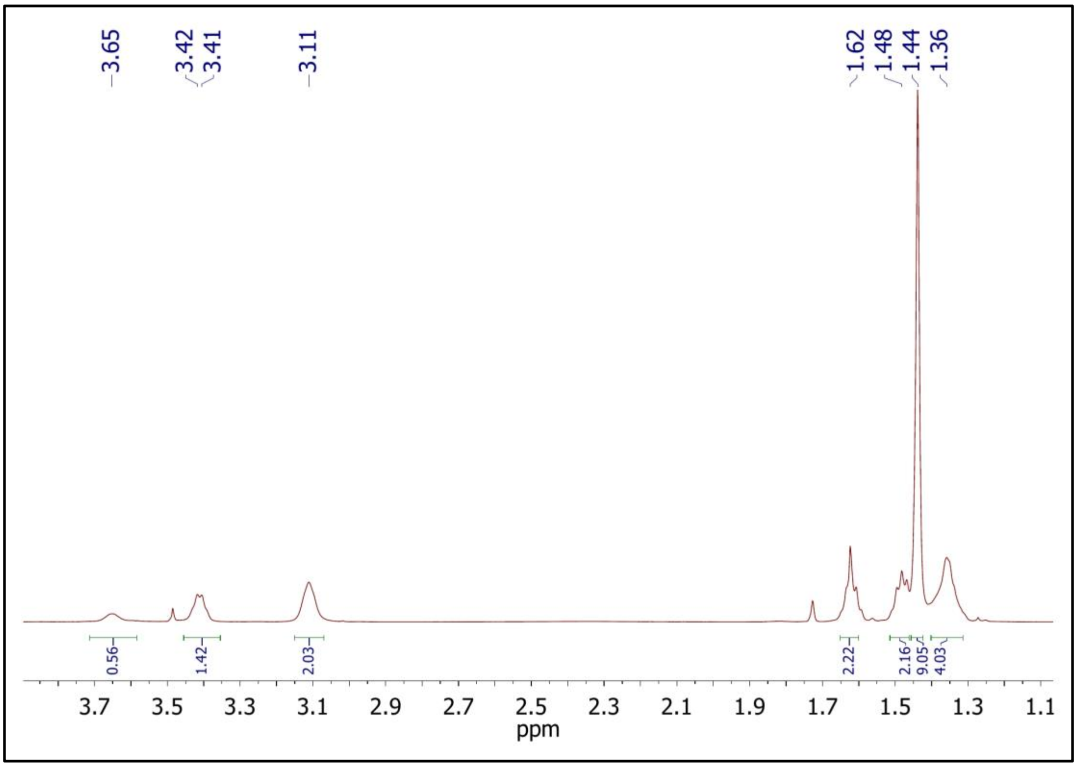

Figure S37c. Lower region of ^{1}H NMR spectrum of **10** (500 MHz, $CDCl_3$)

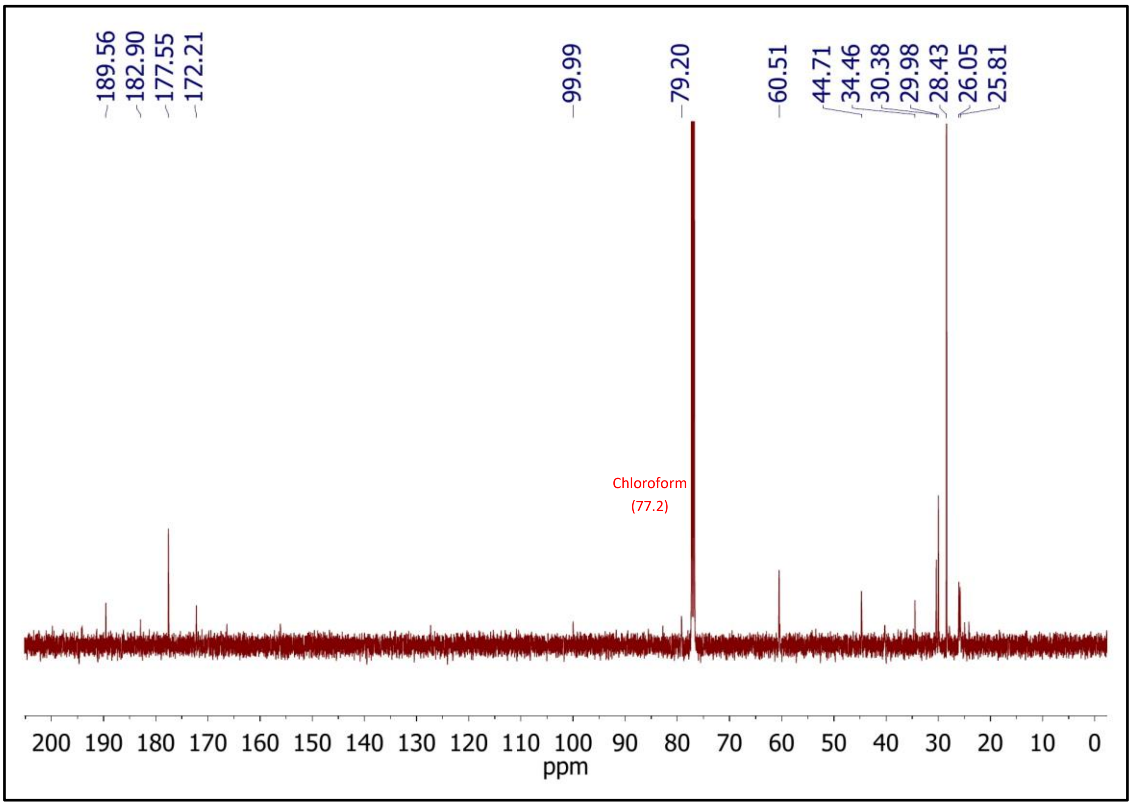

Figure S38. ^{13}C NMR spectrum of **10** (125 MHz, $CDCl_3$)

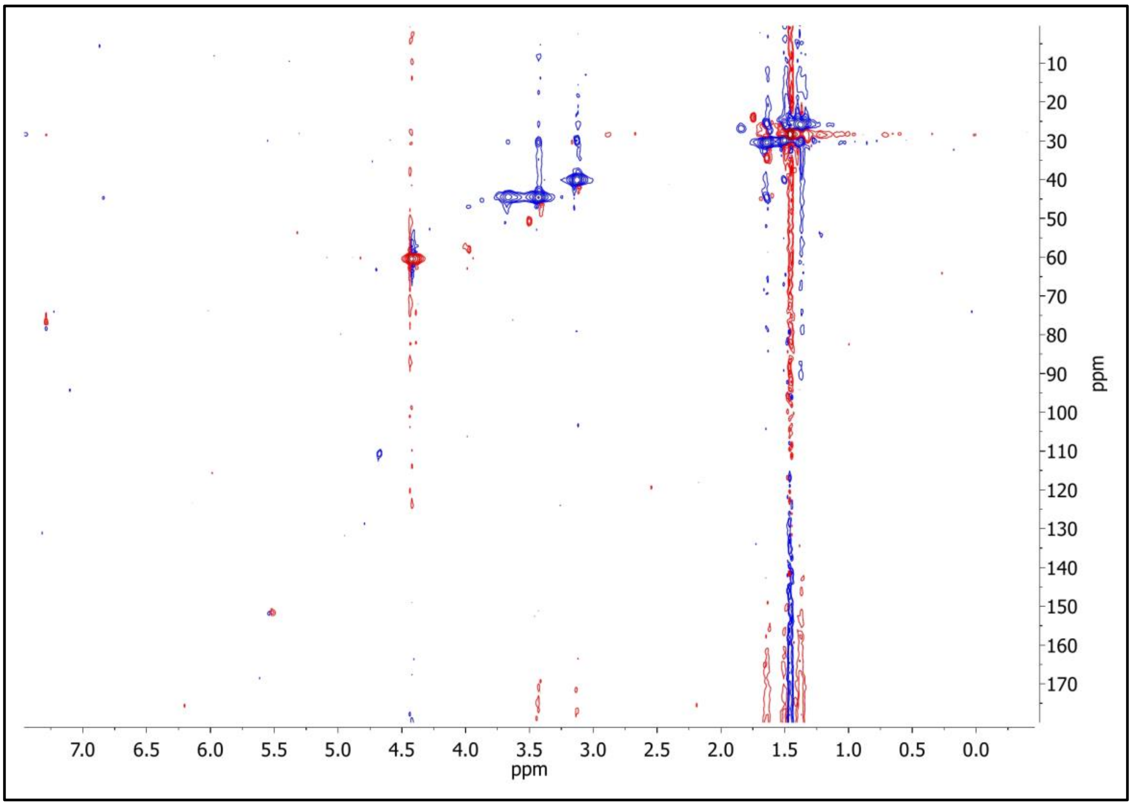

Figure S39. HSQC NMR spectrum of **10** ($CDCl_3$)

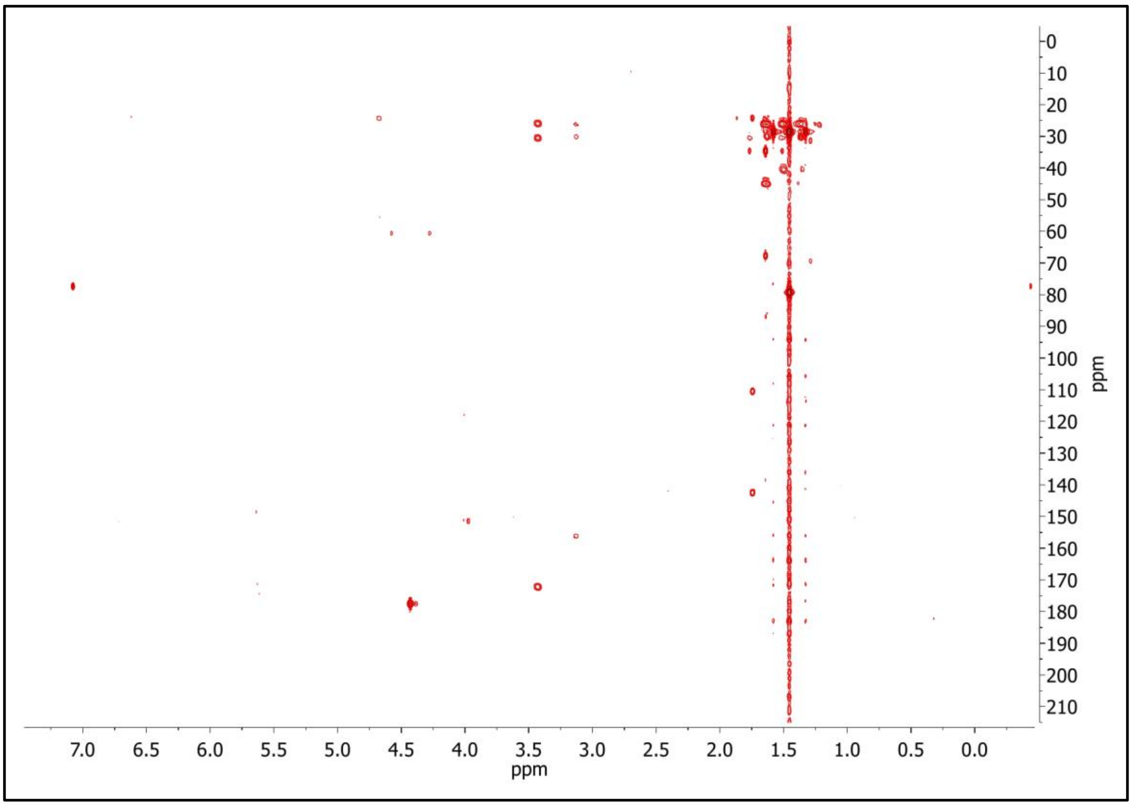

Figure S40. HMBC NMR spectrum of **10** ($CDCl_3$)

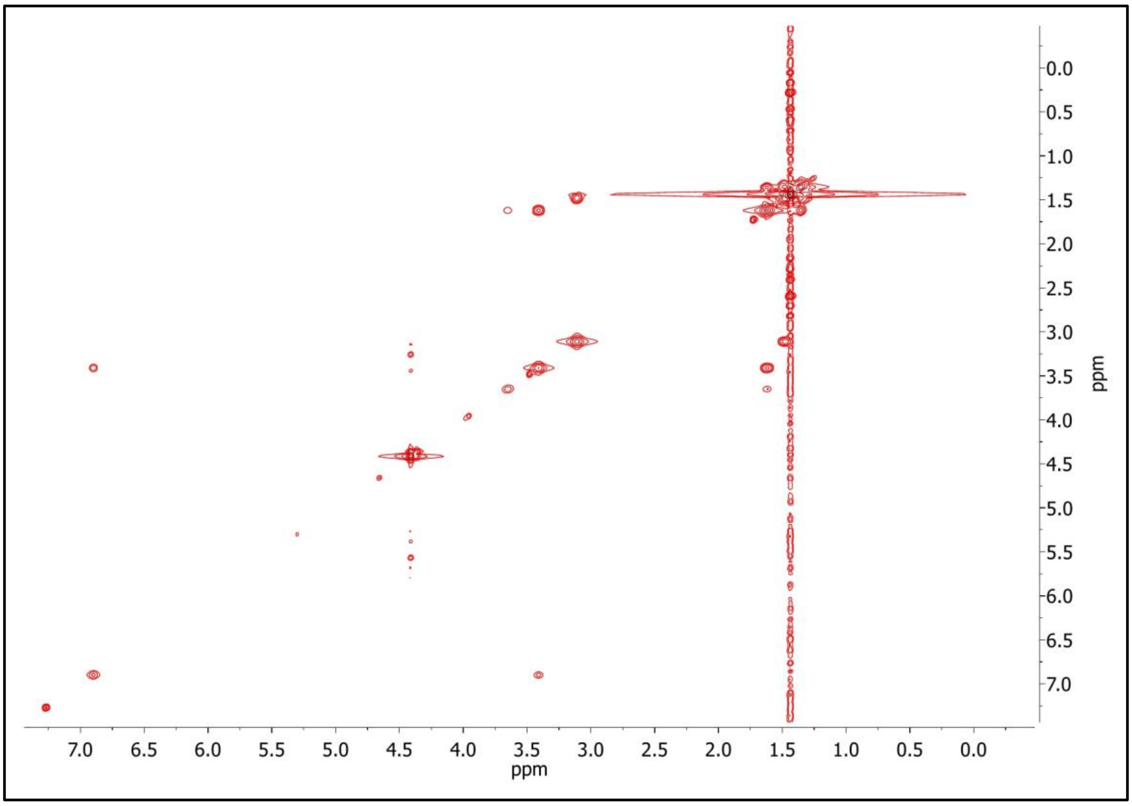

Figure S41. COSY NMR spectrum of **10** ($CDCl_3$)

NMR spectra of **12**

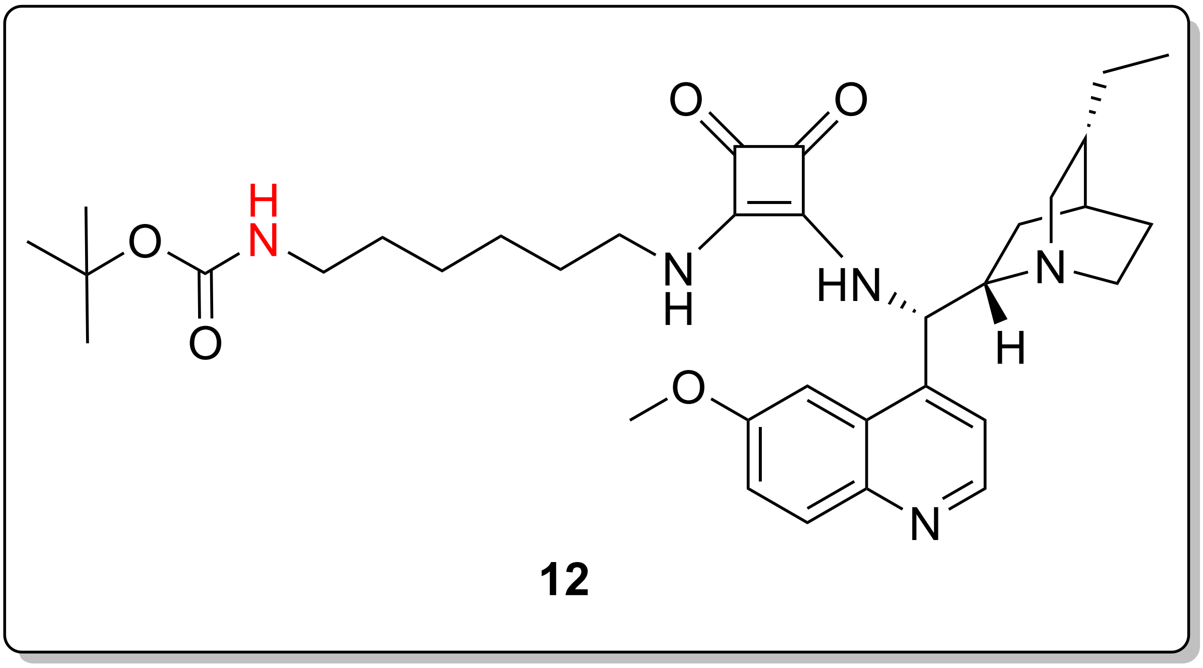

Figure S42. Molecule structure of **12**

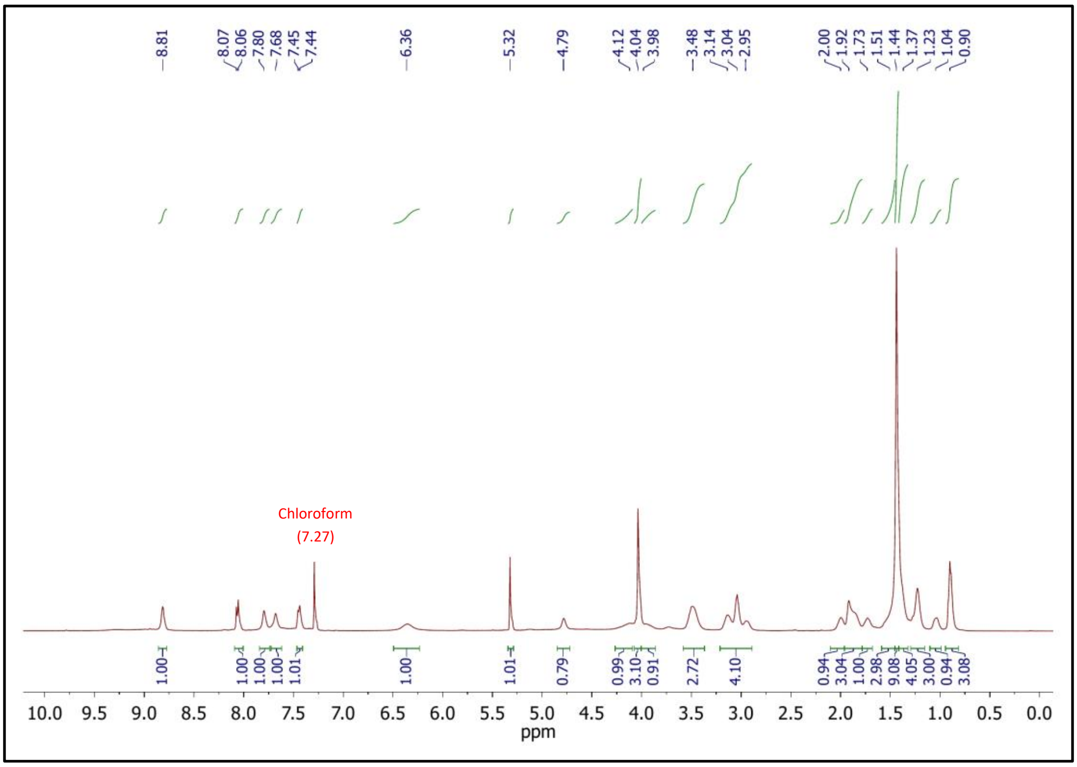

Figure S43. ^{1}H NMR spectrum of **12** (500 MHz, $CDCl_3$)

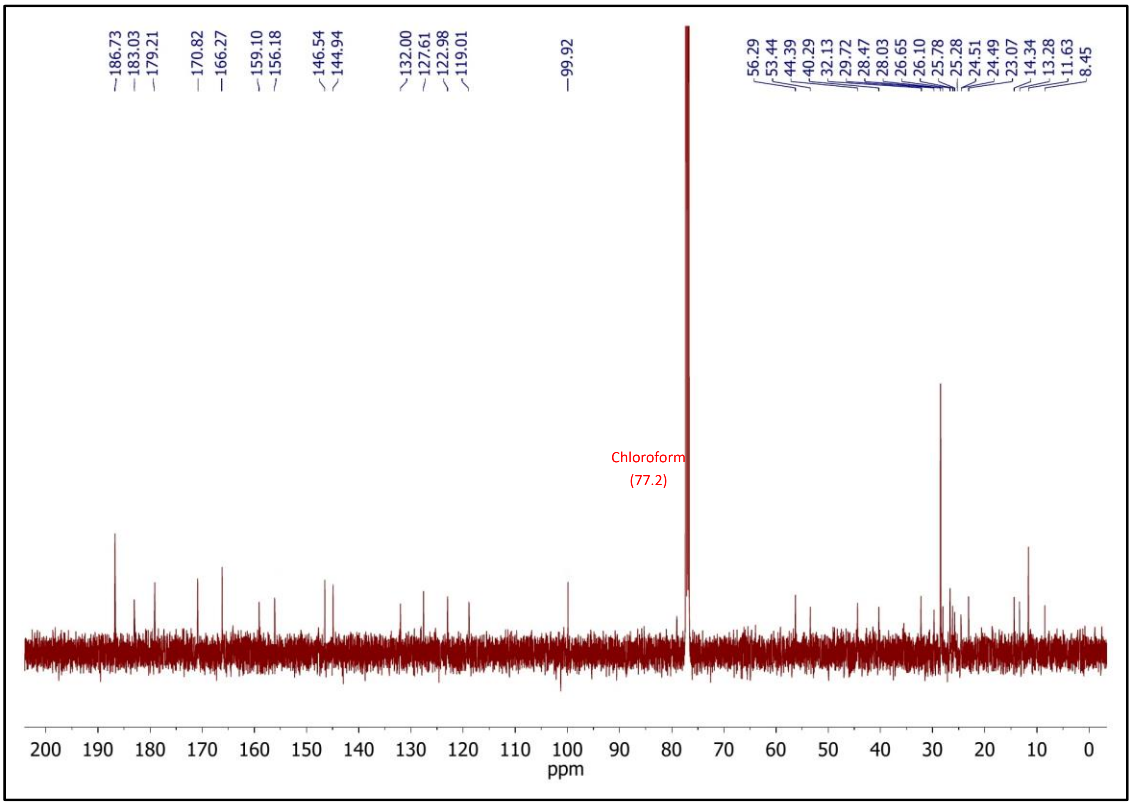

Figure S44. ^{13}C NMR spectrum of **12** (125 MHz, $CDCl_3$)

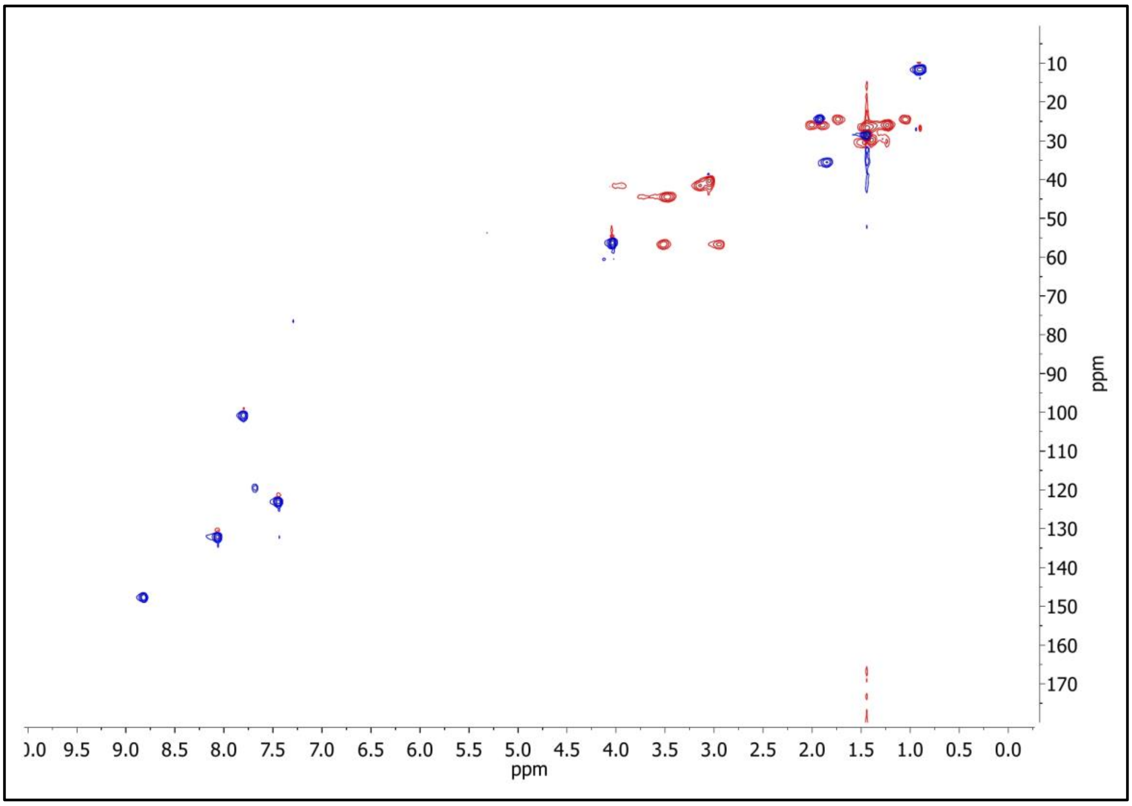

Figure S45. HSQC NMR spectrum of **12** ($CDCl_3$)

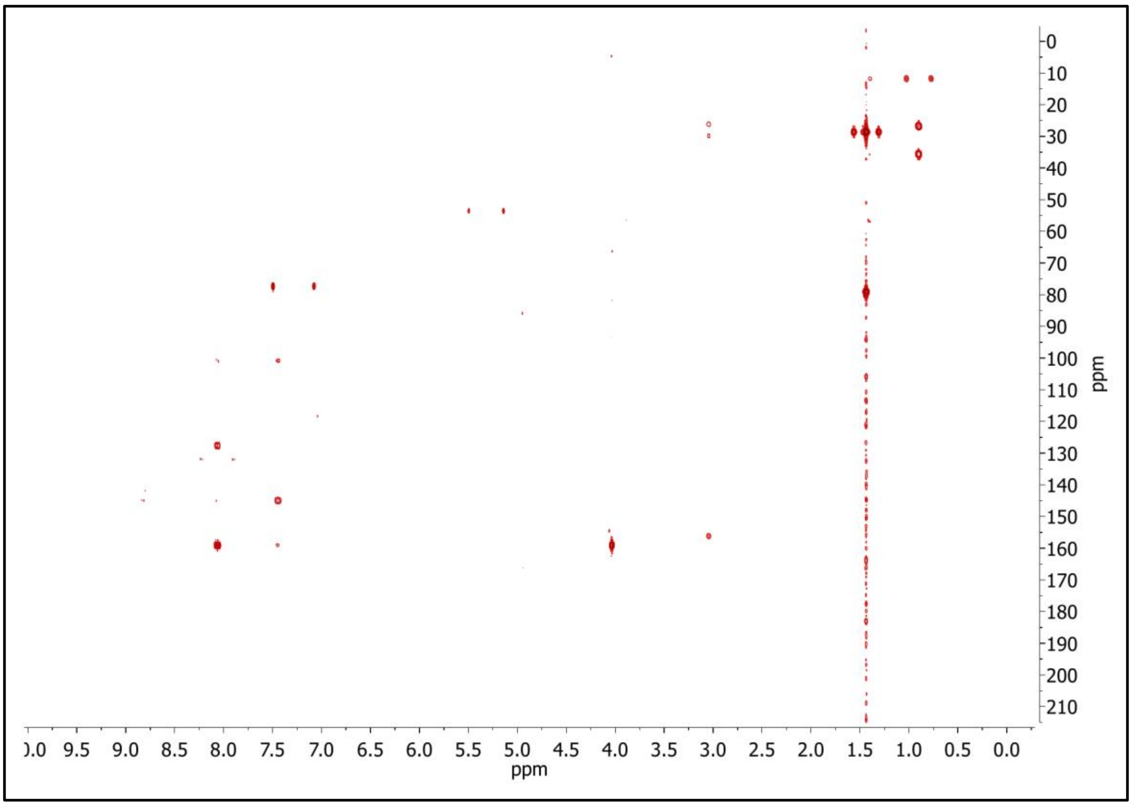

Figure S46. HMBC NMR spectrum of **12** ($CDCl_3$)

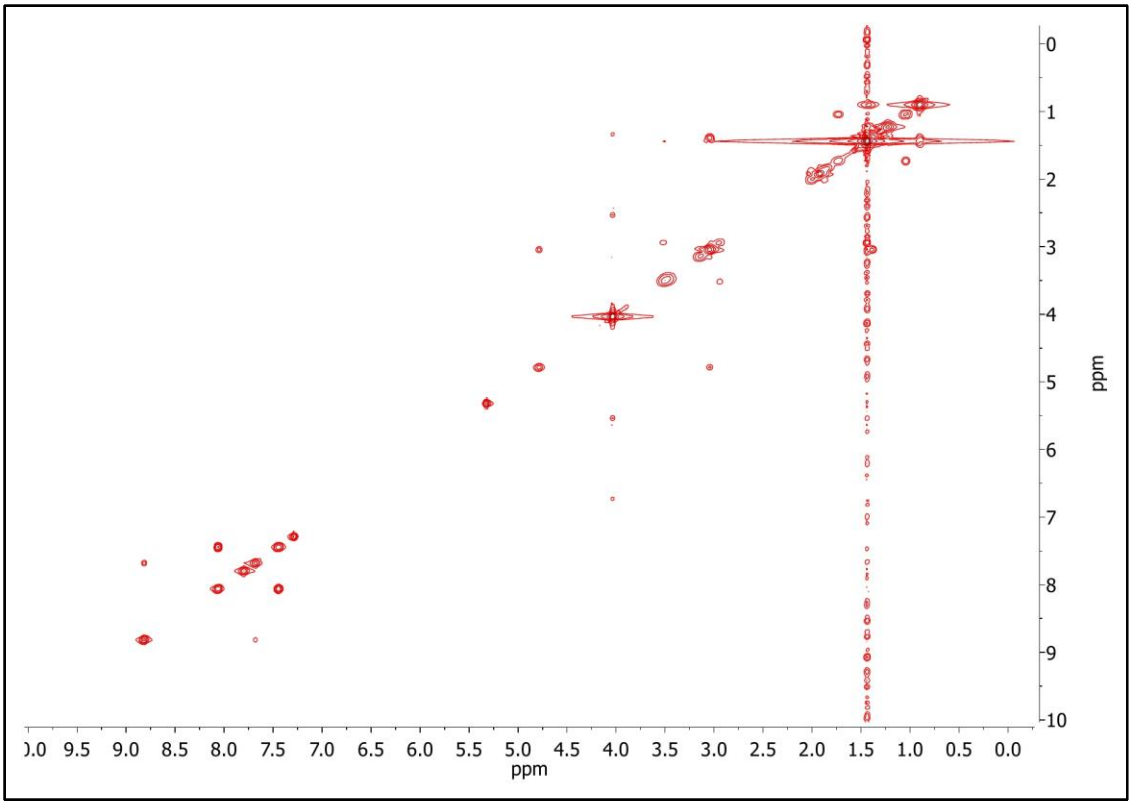

Figure S47. COSY NMR spectrum of **12** ($CDCl_3$)

3, Chiral HPLC profiles of *Michael* adducts **15**

HPLC: Phenomonex Lux Cellulose-1 column (5 µm, 250 × 4.6 mm), eluent hexane:ethanol 85:15, isocratic mode; 0.8 mL min^{-1} ; temperature 20 °C, UV detector 254 nm. Retention time for (*S*)-**15**: 16.1 min, for (*R*)-**15**: 17.6 min.

Peak #	Time [min]	Area [µV·s]	Height [µV]	Area [%]	Norm. Area [%]	BL	Area/Height [s]
1	16.226	658669.45	27157.26	50.95	50.95	*BB	24.2539
2	17.717	634054.32	14296.74	49.05	49.05	*BB	44.3496
		1292723.78	41454.00	100.00	100.00		

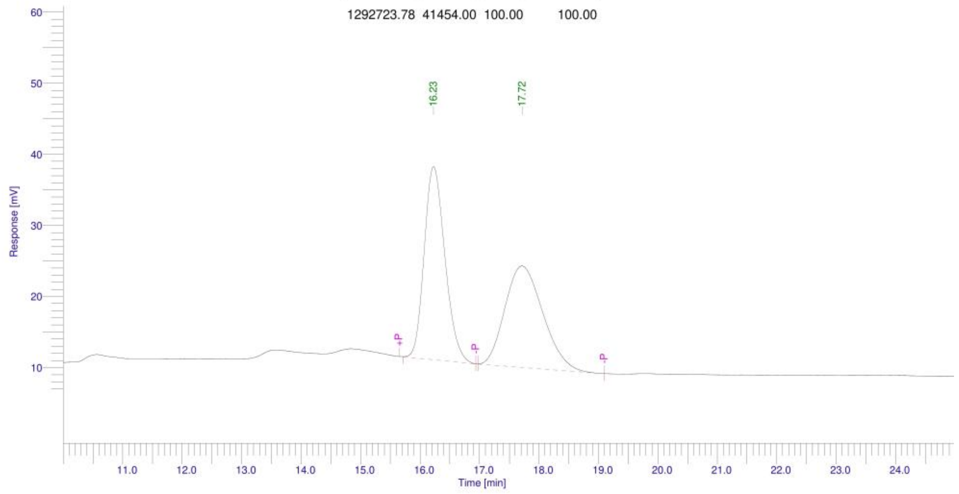

Figure S48. HPLC chromatogram of racemic **15**

Peak #	Time [min]	Area [µV·s]	Height [µV]	Area [%]	Norm. Area [%]	BL	Area/Height [s]
1	16.114	900947.87	36564.47	98.10	98.10	*BB	24.6400
2	17.615	17442.67	452.97	1.90	1.90	*BB	38.5073
		918390.54	37017.44	100.00	100.00		

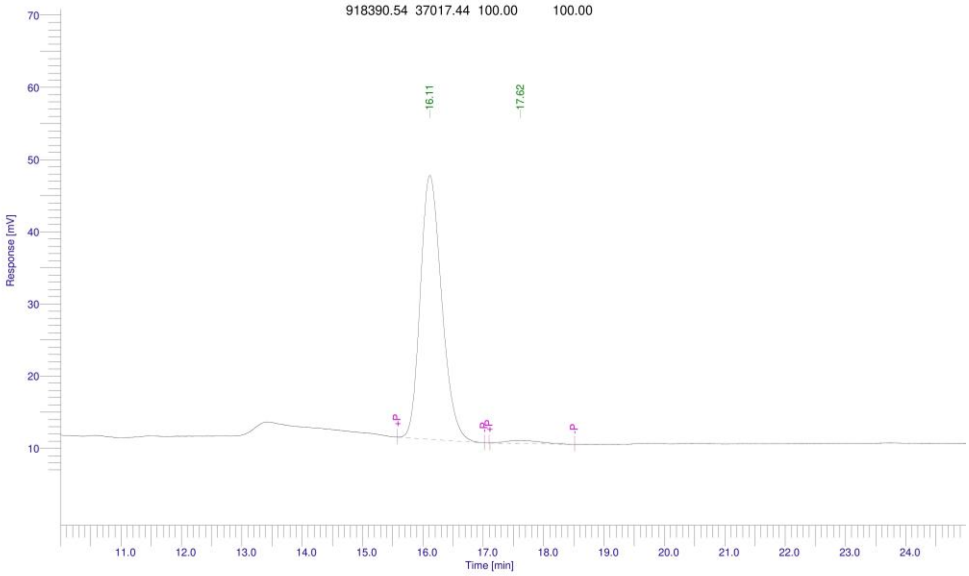

Figure S49. HPLC chromatogram of enantiomeric enriched **15** (Table 4, Round 1)

1. D. Horak, P. Shapoval, *J. Polym. Sci., Part A: Polym. Chem.* **2000**, 38, 3855.
